# Temporal optimization of CD25-biased IL-2 agonists and immune checkpoint blockade leads to synergistic anticancer activity despite robust regulatory T cell expansion

**DOI:** 10.1136/jitc-2024-010465

**Published:** 2025-08-11

**Authors:** Irfan Baki Kilic, Petra Weberova, Derek VanDyke, Milada Sirova, Katerina Kubesova, Charina S Fabilane, Vladyslav Mazhara, Kathy Liu, Katerina Behalova, Bohumil Ptacek, Blanka Rihova, Jamie B Spangler, Marek Kovar

**Affiliations:** 1 Laboratory of Tumor Immunology, Institute of Microbiology of the Czech Academy of Sciences, Prague, Czech Republic; 2 Department of Chemical and Biomolecular Engineering, Johns Hopkins University, Baltimore, Maryland, USA; 3 Translational Tissue Engineering Center, Johns Hopkins University School of Medicine, Baltimore, Maryland, USA; 4 Program in Molecular Biophysics, Johns Hopkins University, Baltimore, Maryland, USA; 5 Department of Biomedical Engineering, Johns Hopkins University School of Medicine, Baltimore, Maryland, USA; 6 Bloomberg~Kimmel Institute for Cancer Immunotherapy, Johns Hopkins University, Baltimore, Maryland, USA; 7 Sidney Kimmel Comprehensive Cancer Center, Johns Hopkins University, Baltimore, Maryland, USA; 8 Department of Oncology, Johns Hopkins University School of Medicine, Baltimore, Maryland, USA; 9 Department of Ophthalmology, Johns Hopkins University School of Medicine, Baltimore, Maryland, USA; 10 Department of Molecular Microbiology & Immunology, Johns Hopkins University Bloomberg School of Public Health, Baltimore, Maryland, USA

**Keywords:** Cytokine, Immune Checkpoint Inhibitor, Immunotherapy, T cell, T regulatory cell - Treg

## Abstract

**Background:**

Interleukin-2 (IL-2) immunotherapy can induce durable tumor remissions, but its clinical performance has been limited by significant drawbacks such as short serum half-life and high toxicity. Administration of IL-2 in complex with certain anti-IL-2 antibodies (IL-2cx) enhances circulation half-life while also selectivity directing the cytokine to particular immune cell subsets. In particular, IL-2cx has been developed that targets either cells expressing the CD25-containing high-affinity IL-2 receptor (ie, CD25-biased IL-2cx) or cells expressing the CD25-lacking intermediate-affinity IL-2 receptor (ie, CD25-blocking IL-2cx). Since regulatory T (Treg) cells primarily express the high-affinity IL-2 receptor whereas naïve effector T and natural killer cells mainly express the low-affinity IL-2 receptor, CD25-blocking IL-2cx have traditionally been considered as potential cancer therapeutics, particularly in combination with immune checkpoint inhibitors (ICIs).

**Methods:**

Stimulation of antigen-primed T cells by IL-2cx in the absence or presence of ICIs was evaluated through adoptive transfer of primed ovalbumin-specific T cells and analysis of expansion. Effects of IL-2cx on Treg cell-mediated inhibition of CD8^+^ T cells were assessed by flow cytometry and thymidine incorporation. Tumor-bearing mice received combination treatments comprizing IL-2cx and ICIs, where complexes were delivered either before or after ICIs. Tumor growth and mouse survival were monitored, and immune cell phenotyping was performed. Toxicity was determined by tracking body weight, temperature, and lung edema. Substitution of IL-2cx with single-agent cytokine/antibody fusion proteins (immunocytokines, ICs) was also explored.

**Results:**

We showed that CD25-biased IL-2cx and ICs synergize with ICIs to completely eradicate large, established tumors despite robust Treg cell expansion. Importantly, we found that timing is crucial, as administration of IL-2cx after (but not before) ICIs led to profound antitumor effects. Mechanistically, CD25-biased IL-2cx selectively stimulated expansion and effector functions of tumor-specific CD8^+^ T cells in a CD25-dependent manner, overcoming Treg cell-mediated suppression. Moreover, CD25-biased IL-2cx showed much lower toxicity than CD25-blocking IL-2cx, enabling a larger therapeutic window. Furthermore, we demonstrated that administration of a human IL-2-based IC significantly enhanced the antitumor activity of ICIs, establishing the translational relevance of our work.

**Conclusions:**

Our findings support the temporally optimized use of CD25-biased IL-2-based therapeutics in combination with ICIs for cancer immunotherapy.

**WHAT IS ALREADY KNOWN ON THIS TOPIC**

- Efforts in the field of interleukin-2 (IL-2)-based cancer therapeutic design have focused on approaches, including cytokine/antibody complexes (IL-2cx) or cytokine/antibody fusion proteins (immunocytokines (ICs)), cytokine mutants, fusion proteins, and polymer conjugates, that selectively stimulate cells expressing the intermediate-affinity IL-2 receptor (ie, memory phenotype CD8^+^ T and natural killer cells). Such IL-2-based therapeutics have shown anti-cancer activity across a range of preclinical models.

**WHAT THIS STUDY ADDS**

- This study demonstrates that IL-2cx or ICs that selectively stimulate cells expressing the high-affinity IL-2 receptor (CD25-biased IL-2cx) lead to profound antitumor activity in the absence of toxicity. Importantly, our work is the first to establish that the timing of IL-2 agonist administration relative to immune checkpoint inhibitors (ICIs) is critical for therapeutic efficacy. Moreover, we elucidate the mechanistic basis for these findings, demonstrating that IL-2cx or ICs that are biased towards cells that express the high-affinity IL-2 receptor potently activate recently antigen-primed CD8^+^ T cells and overcome regulatory T cell-mediated suppression at saturating levels. Finally, we provide direct comparisons between CD25-biased and CD25-blocking IL-2cx in terms of efficacy, toxicity, and immune activation profiles, and extend our findings using translationally relevant human ICs.

**HOW THIS STUDY MIGHT AFFECT RESEARCH, PRACTICE OR POLICY**

- Our study establishes an effective strategy for using IL-2cx or ICs that selectively stimulate cells expressing the high-affinity IL-2 receptor to boost the antitumor efficacy of ICIs, presenting a promising new paradigm for cancer immunotherapy.

## Introduction

 Interleukin-2 (IL-2) is a multifunctional cytokine predominantly produced by activated T cells.^1^ IL-2 exerts its pleiotropic activities through binding to either an intermediate-affinity dimeric receptor composed of the IL-2 receptor beta (IL-2Rβ, CD122) subunit and the common gamma chain (γ_c_, CD132) with K_D_∼10^−9^ M, or to a high-affinity trimeric receptor composed of the non-signaling IL-2Rα (CD25) subunit, CD122, and CD132 with K_D_∼10^−11^ M.^1 2^ IL-2 engagement of CD122 and CD132 in either the dimeric or trimeric IL-2R leads to activation of the Janus kinase-signal transducer and activator of transcription signaling pathway, which regulates gene expression and mediates the physiologic activities of IL-2 at the molecular and cellular levels.^1^,^3^ CD122^high^ populations, such as natural killer (NK) cells, NK T cells, and resting or memory effector T cells, express low levels of CD25 and are therefore predominantly activated by IL-2 through the dimeric IL-2R. In contrast, CD25 is constitutively high on regulatory T (Treg) cells and is upregulated on recently activated T cells; thus, these immune cell subsets are predominantly activated by IL-2 through the trimeric IL-2R and are significantly more sensitive to IL-2. IL-2 plays a key role in promoting the proliferation, survival, and effector functions of T and NK cells.^1 3^ However, IL-2 is also essential for Treg cell homeostasis and suppressor activity.^3^ Due to their primary utilization of the trimeric IL-2R, Treg cells outcompete NK and effector T cells for IL-2, which serves as an important mechanism through which Treg cells suppress effector T cell-mediated immune responses.^4^

IL-2 was the first Food and Drug Administration-approved immunotherapeutic for metastatic renal cell carcinoma and malignant melanoma.^5^ IL-2 has demonstrated moderate efficacy in these cancers, with complete response (CR) rates of 9.3% and 4%, respectively, and a significant portion of these CRs has been long-term durable remissions.^6^ However, IL-2 is only effective against cancer at high doses, due to its weaker interaction with dimeric IL-2R-expressing NK and effector T cells compared with trimeric IL-2R-expressing Treg cells, as well as the cytokine’s extremely short half-life. Unfortunately, high-dose IL-2 therapy leads to severe toxicities, including vascular leak syndrome, which can lead to pulmonary edema, organ failure, and death.^7^ Insufficient efficacy combined with its considerable toxicity has thus prevented widespread use of IL-2 in cancer treatment. Moreover, the concomitant expansion of Treg cells on IL-2 therapy also raises questions as to its suitability for cancer immunotherapy.^8^ Indeed, low-dose IL-2 therapy, which selectively expands and activates Treg cells without stimulating T and NK cells, has proven effective in suppressing the immune response for the treatment of several autoimmune diseases, such as type 1 diabetes.^2 9^

Numerous studies have described the improvement of IL-2 therapy in cancer through engineering approaches, including the design of IL-2 muteins with biased cytokine activity (reducing CD25 binding and/or increasing CD122 binding),^8^polyethylene glycol (PEG)ylation of IL-2 and variants thereof^10^ or design of IL-2/CD25 fusion proteins (FPs).^11^ Another interesting approach, first described by Boyman *et al*,^12^ uses complexes of IL-2 with an anti-IL-2 antibody (IL-2cx). IL-2cx possesses dramatically increased in vivo biological activity compared with the cytokine alone, due to both extension of IL-2 half-life through neonatal Fc receptor-mediated recycling^2 13^ as well as structure-based modulation of IL-2 activity.^2 14^ IL-2 complexed with the antibody S4B6 (IL-2/S4B6 henceforth) was shown to preferentially stimulate NK and resting/memory effector T cell populations via dimeric IL-2R, whereas IL-2 complexed with the antibody JES6-1A12 (IL-2/JES6 henceforth) selectively stimulates CD25^high^ populations (ie, Treg cells) via trimeric IL-2R.^12^,^15^ Notably, IL-2/S4B6 also stimulates CD25^high^ populations to some extent, although in a CD25-independent manner.^14 15^ Mechanistically, it has been shown that IL-2/S4B6 directly binds to dimeric IL-2R while completely avoiding interaction with CD25 due to overlapping binding sites for CD25 and the S4B6 antibody on the IL-2 cytokine.^2 14^ On the other hand, activation of trimeric IL-2R by IL-2/JES6 requires cytokine interaction with CD25 in the first step, followed by dissociation of IL-2 from the JES6-1A12 antibody, and subsequent engagement of the full trimeric IL-2R.^2 14^ Thus, IL-2/JES6 is biased towards activation of CD25^high^ cells, which leads to preferential Treg stimulation. Notably, effector T cell populations also upregulate CD25 on activation and can therefore also respond to IL-2/JES6 stimulation.^15^ To stabilize the IL-2/anti-IL-2 interaction within IL-2cx, single-chain FPs known as immunocytokines (ICs) were developed, where IL-2 is connected through a flexible oligopeptide linker to the N-terminus of the light chain of the anti-IL-2 antibody.^16 17^ ICs mimic IL-2cx both structurally and functionally but prevent dissociation of the cytokine while also fixing the stoichiometric ratio, thereby extending half-life, limiting off-target effects, and presenting a clinically relevant format.^18^

IL-2/S4B6 has been shown to induce potent stimulatory activity of NK cells as well as memory and activated CD8^+^ T cells, and since the complex has demonstrated antitumor activity in several mouse tumor models, it has been established for more than a decade that these CD25-blocking IL-2cx are suitable for cancer immunotherapy.^2 15^ Meanwhile, the CD25-biased IL-2/JES6 has been shown to extensively expand Treg cells and has been effective in preclinical models of autoimmune disease^2 19^ and transplantation.^20^ However, several recent studies have shown that targeting the high-affinity trimeric IL-2R may be beneficial in cancer immunotherapy and that CD25-biased IL-2 agonists^21 22^ are more effective antitumor agents in comparison to CD25-blocking IL-2 agonists. Moreover, CD25-binding was found to be indispensable for modifying CD8^+^ T cell exhaustion programs when using IL-2 in combination with a class of immunotherapies known as immune checkpoint inhibitor antibodies (ICIs) in a model of chronic viral infection.^23^ ICIs affect antitumor activity by blocking ligand-receptor interactions that inhibit T cell activation, thereby relieving tumor-promoting immunosuppression.^24^ The use of ICIs in clinical practice, particularly anti-cytotoxic T-lymphocyte-associated antigen 4 (CTLA-4) and anti-programmed cell death 1 (PD-1) antibodies, has revolutionized cancer immunotherapy and has profoundly enhanced treatment of metastatic disease across cancer types (eg, melanoma and lung carcinoma).^24^,^26^ Unfortunately, only a minority (<30%) of patients respond to ICI therapy, and some cancer types resist ICI therapy entirely; thus, there is an urgent need to identify synergistic combination therapies to improve overall response rates^26^ and IL-2-based drugs have shown promising preclinical anticancer activity when combined with ICI therapies.^27 28^

Although IL-2/JES6 has not been extensively tested in tumor models due to its biased expansion of Treg cells, we recently showed that IL-2/JES6 considerably improved doxorubicin-based chemotherapy in the mouse syngeneic B16F10 melanoma and BCL1 leukemia models.^29^ Furthermore, IL-2/JES6 increased the counts of CD49d^−^KLRK1^+^CD8^+^ T cells (termed KILR T cells), many of which express heightened levels of CD127 and granzyme B and possess high cytotoxic potential.^29^ We hypothesized that doxorubicin induced immunogenic cancer cell death, resulting in presentation of tumor antigens to prime tumor-specific T cells. The activated T cells would in turn upregulate CD25, resulting in selective activation of tumor antigen-specific T cells by the CD25-biased IL-2/JES6. Building on this unexpected finding, we asked whether IL-2cx could be used to potentiate cancer immunotherapy in the context of ICI treatment, and whether CD25-biased or CD25-blocking IL-2cx would lead to superior antitumor activity when combined with ICIs.

## Results

### CD25-biased IL-2/JES6 is highly efficient and selective in driving expansion of antigen-primed CD8^+^ T cells

**Figure 1:**
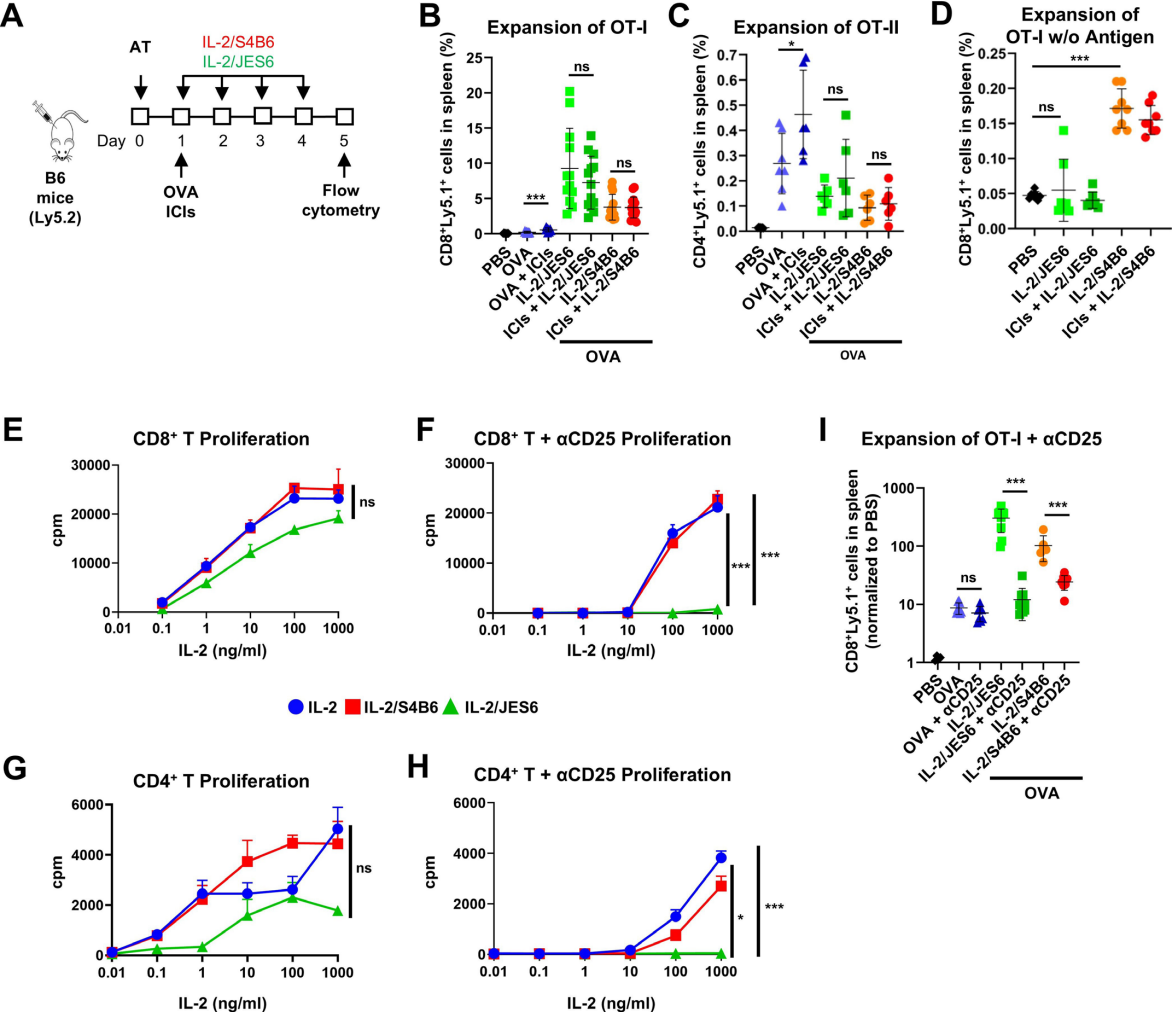
IL-2/JES6 potently and selectively expand antigen-primed CD8^+^ T cells. (**A–C**) Purified CD8^+^ or CD4^+^ T cells from OT-I or OT-II/RAG1^−/−^/Ly5.1 mice, respectively, were adoptively transferred (AT) into B6 mice. Mice were i.p. injected with 350 µg OVA, ICIs (αCTLA-4 + αPD-1 antibodies; 0.5 mg/kg each) and IL-2cx (2 µg IL-2/dose) and their spleens were analyzed by flow cytometry. A schematic of the study is shown (**A**), as is expansion of AT CD8^+^ (**B**) and CD4^+^ T (**C**) cells with depicted average±SD for all experimental groups. Each point represents an individual mouse. Data pooled from two to three independent experiments with n=6–12. (**D**), Purified CTV-labeled CD8^+^ OT-I cells were AT into B6 mice. Treatment was as depicted in (**A**), except the mice were not injected with OVA. Expansion of CD8^+^ T cells with depicted average±SD for all experimental groups. Each point represents an individual mouse. Data pooled from two independent experiments with n=6–8. (**E-H**), Proliferation of naive CD8^+^ (**E, F**) and CD4^+^ (**G, H**) T cells incubated in αCD3 antibody-coated wells with titrated concentrations of IL-2 or IL-2cx and αCD25 antibody (**F, H**) was determined by [^3^H]-thymidine incorporation. Data shown as average cpm from triplicates±SD. Experiment was conducted twice with comparable results. Results were statistically analyzed using an unpaired t-test for the highest concentration (ns: non-significant; *p<0.05; ***p<0.001). (**I**), Purified CTV-labeled CD8^+^ OT-I cells were AT into B6 mice. Treatment was as depicted in (**A**) except the mice were not injected with ICIs but i.p. with αCD25 antibody (200 µg/dose) on days 1 and 3. Expansion of CD8^+^ T cells with depicted average±SD for all experimental groups is shown. Each point represents an individual mouse. Data pooled from two independent experiments with n=6–8. Results were statistically analyzed by unpaired t-test (ns: non-significant; *p<0.05; ***p<0.001). αCTLA-4, anti-cytotoxic T-lymphocyte-associated antigen 4; αPD-1, anti-programmed cell death 1; cpm, counts per minute; CTV, CellTrace Violet; ICIs, immune checkpoint inhibitors; IL-2, interleukin-2; IL-2cx, anti-IL-2 antibodies; i.p., intraperitoneally; OVA, ovalbumin; PBS, phosphate-buffered saline.

To evaluate the potential of CD25-blocking versus CD25-biased IL-2cx to promote the expansion of antigen-primed T cells, we adoptively transferred OT-I CD8^+^ T cells or OT-II CD4^+^ T cells into congenic mice and primed them with ovalbumin (OVA) in the presence or absence of ICIs (anti-CTLA-4 plus anti-PD-1 antibodies). Mice were subsequently treated with either the CD25-biased IL-2/JES6 or the CD25-blocking IL-2/S4B6 and their splenocytes were analyzed by flow cytometry (figure 1A). Both IL-2cx increased the expansion of OVA-primed OT-I CD8^+^ T cells; however, IL-2/JES6 led to approximately ∼2.5 times greater expansion than IL-2/S4B6 (figure 1B, online supplemental figure 1A-upper row). Administration of ICIs augmented the expansion of OT-I CD8^+^ T cells stimulated with OVA alone but not those stimulated with OVA plus either IL-2cx. Interestingly, neither IL-2cx increased the expansion of OVA-primed OT-II CD4^+^ T cells (figure 1C, online supplemental figure 1A-bottom row). In the absence of antigen priming, IL-2/S4B6 but not IL-2/JES6 stimulated the proliferation and expansion of naïve OT-I CD8^+^ T cells (figure 1D, online supplemental figure 1B and C). Moreover, both IL-2cx dramatically increased levels of the proliferation marker Ki-67, expression of CD25, granzyme B levels, and perforin levels in expanded OT-I CD8^+^ T cells (online supplemental figure 2A and B). Increased expression of these molecules was also observed in endogenous CD8^+^ T cells (online supplemental figure 2C and D) and in the absence of OVA stimulation (online supplemental figure 2E and F). Collectively, these studies demonstrate that IL-2/JES6 potently and selectively stimulate expansion of antigen-primed CD8^+^ but not CD4^+^ T cells. In addition to their activities on effector T cells, IL-2/JES6 significantly expanded Treg cells (online supplemental figure 2G), while IL-2/S4B6 potently boosted NK cell abundance (online supplemental figure 2H), as expected.

To determine whether these in vivo effects were recapitulated in vitro, primary mouse T cells were activated by plate-bound anti-CD3 antibody and subsequently stimulated with IL-2cx. In this context, proliferation of CD8^+^ T cells was somewhat lower in response to IL-2/JES6 compared with IL-2 alone or IL-2/S4B6 (figure 1E). Furthermore, CD8^+^ T cell growth was attenuated in the presence of an anti-CD25 antibody following treatment with IL-2 alone or IL-2/S4B6 and completely abrogated following treatment with IL-2/JES6 (figure 1F), indicating that IL-2/JES6-induced proliferation relied exclusively on trimeric IL-2R signaling. IL-2 alone, IL-2/S4B6, and IL-2/JES6 induced CD4^+^ T cell growth in vitro (figure 1G), contrasting with the lack of IL-2cx-mediated expansion of CD4^+^ T cells in vivo. However, in vitro proliferation of CD4^+^ T cells was much weaker than that of CD8^+^ T cells. Notably, increased proliferation for cells treated with IL-2/S4B6 compared with those treated with IL-2/JES61 was more pronounced for CD4^+^ T cells versus CD8^+^ T cells (figure 1E and G). In the presence of an anti-CD25 antibody, IL-2-induced and IL-2/S4B6-induced proliferation of CD4^+^ T cells was attenuated, whereas IL-2/JES61-induced proliferation of CD4^+^ T cells was completely vitiated (figure 1H). In the context of antigen-primed CD8^+^ T cells, CD25 blockade partially reduced the stimulatory effect of IL-2/S4B6 and completely eliminated the stimulatory effect of IL-2/JES61 (figure 1I, online supplemental figure 1D and E), again demonstrating the dependence of IL-2/JES6-induced expansion on trimeric IL-2R activity. Partial inhibition of IL-2/S4B6 effects by an anti-CD25 antibody (figure 1F, H1, online supplemental figure 1D and E) was observed despite the fact that the S4B6 antibody neutralizes IL-2 interactions with CD25, consistent with previous findings.^14^ Collectively, in vitro and in vivo proliferation studies demonstrated that IL-2cx stimulates expansion of naïve and antigen-experienced T cells in both the presence and absence of ICI treatment, although the effects differ between T cell subsets and for distinct IL-2/antibody complexes.

### Treg cells do not suppress CD8^+^ T cells when sufficient amounts of IL-2/JES6 are available

Although IL-2/JES6 was found to stimulate the expansion of activated CD8^+^ T cells, its administration also significantly expanded Treg cells (online supplemental figure 2G), as previously reported.^12 15^ Thus, we investigated whether IL-2/JES6 administration could lead to Treg cell-mediated inhibition of CD8^+^ T cells. It has been established that one of the key mechanisms through which Treg cells inhibit CD8^+^ T cells is by limiting availability of IL-2.^30^ Thus, we hypothesized that in the presence of sufficient IL-2/JES6, Tregs would not impede CD8^+^ T cell activity.

**Figure 2:**
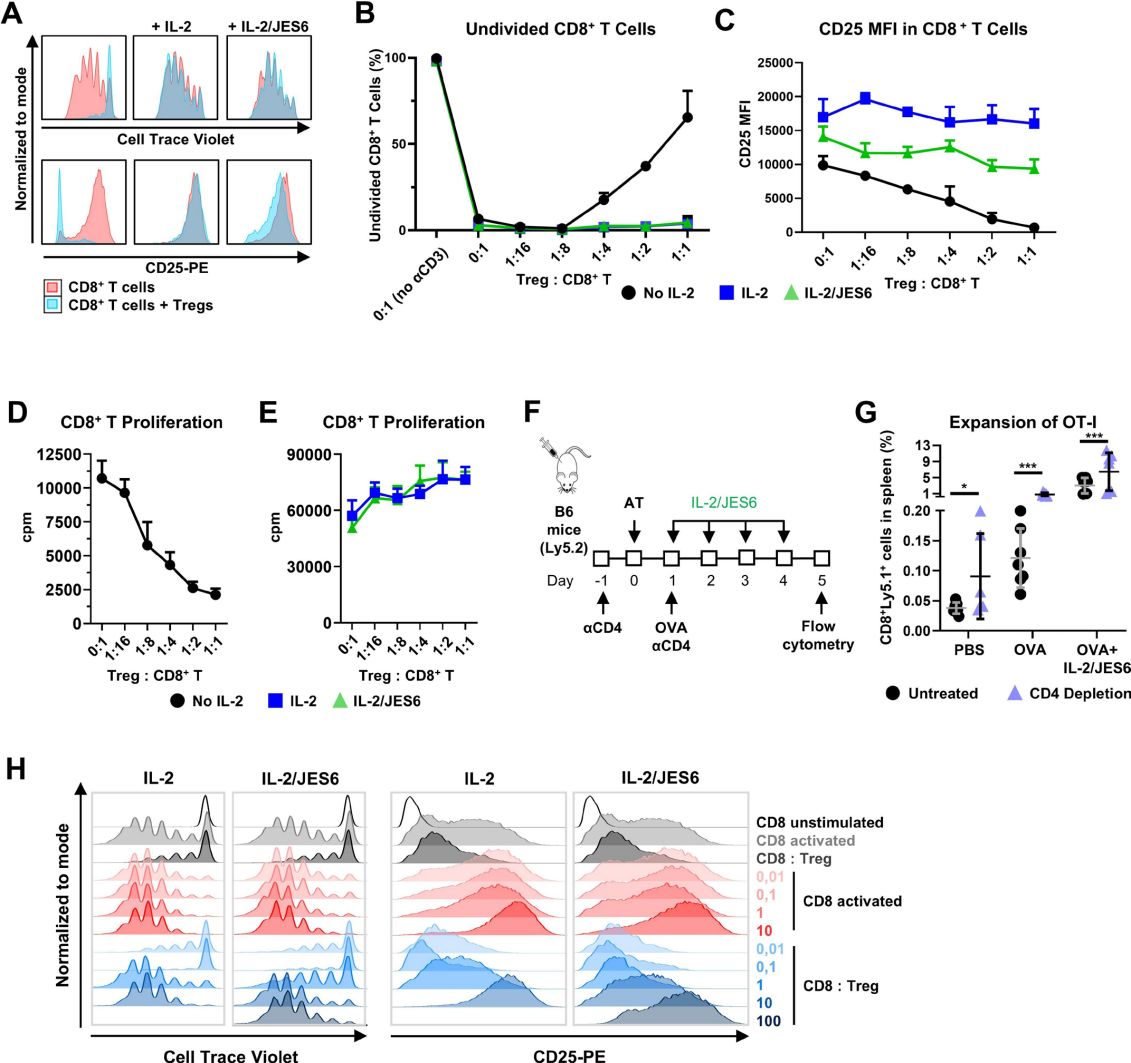
IL-2/JES6 overcomes Treg cell-mediated suppression of CD8^+^ T cells. (**A–C**) CTV-labeled CD8^+^ T cells were cocultivated with Treg cells at the indicated ratios in αCD3 antibody-coated wells alone, with IL-2, or with IL-2/JES6 (10 ng IL-2/mL) for 72 hours. Overlay plots of CTV and CD25 expression for a CD8^+^:Treg cell ratio 1:1 versus CD8^+^ T cells only are shown (**A**), as is relative content of undivided CD8^+^ T cells in each experimental condition±SD (**B**) and average MFI of CD25 in CD8^+^ T cells±SD (**C**). (**D–E**) CD8^+^ T cells were cocultivated with Treg cells at the indicated ratios in αCD3 antibody-coated wells alone, with IL-2, or with IL-2/JES6 (10 ng IL-2/mL) for 72 hours. Proliferation of CD8^+^ T cells was determined by [^3^H]-thymidine incorporation, with data depicted as average cpm±SD for cells incubated in the absence of IL-2 (**D**) or with IL-2 or IL-2/JES6 (**E**). Experiments were performed three times with similar results. (**F-G**), Purified CD8^+^ from OT-I/RAG1^−/−^/Ly5.1 mice were adoptively transferred (AT) into B6 mice. Mice were i.p. injected with 350 µg OVA, αCD4 antibody (200 µg/dose) and IL-2/JES6 (8 µg IL-2/dose) and their spleens were analyzed by flow cytometry. A schematic of the study is shown (**F**), as is expansion of AT CD8^+^ T cells with depicted average±SD for all experimental groups (**G**). Each point represents an individual mouse. Data pooled from two independent experiments with n=7. Results were statistically analyzed by unpaired t-test (*p<0.05; ***p<0.001). (**H**) CTV-labeled CD8^+^ T cells were co-cultured with Treg cells at a 1:1 ratio in αCD3 antibody-coated wells, either alone or in the presence of titrated concentrations of IL-2 or IL-2/JES6 (0.01–100 ng IL-2/mL; red and blue) for 72 hours. Overlay plots depict CTV dilution (indicative of cell proliferation) and CD25 expression; cpm, counts per minute; CTV, CellTrace Violet; IL-2, interleukin-2; i.p., intraperitoneally; MFI, mean fluorescence intensity; OVA, ovalbumin; PBS, phosphate-buffered saline; Tregs, regulatory T cells.

We cocultivated naïve CellTrace Violet (CTV)-labeled CD8^+^ T cells with Treg cells in anti-CD3 antibody-coated wells alone or in the presence of either IL-2 or IL-2/JES6 (10 ng IL-2/ml) and analyzed CD8^+^ T cell proliferation and CD25 expression by flow cytometry. We found that treating with either IL-2 or IL-2/JES6 completely abrogated the potential of Treg cells to suppress the in vitro proliferation of activated CD8^+^ T cells (figure 2A–B, online supplemental figure 3A). Moreover, both IL-2 and IL-2/JES6 prevented Treg-mediated inhibition of CD25 expression on CD8^+^ T cells (figure 2C, online supplemental figure 3B). The capacity of IL-2/JES6 to overcome the Treg cell-mediated inhibition of activated CD8^+^ T cell proliferation was confirmed by [^3^H]-thymidine incorporation (figure 2D and E). To verify these findings in vivo, we adoptively transferred OT-I CD8^+^ T cells into either normal or CD4^+^ T cell-depleted congenic mice. Mice were primed with OVA in the absence or presence of IL-2/JES6 treatment (figure 2F). The expansion of OT-I CD8^+^ T cells was ∼6 times higher in CD4^+^ T cell-depleted acceptors (ie, in the absence of Treg cells) compared with non-depleted acceptors in the context of OVA priming only (figure 2G). By contrast, the difference in expansion was two times lower in OVA-primed mice that received IL-2/JES6, demonstrating that Treg cell-mediated suppression of CD8^+^ T cell expansion was considerably attenuated in the context of IL-2/JES6 delivery in vivo. To further validate our hypothesis that competition for IL-2 between activated CD8^+^ effector T cells and Treg cells leads to Treg-mediated suppression, we performed a Treg suppression assay using titrated concentrations of IL-2 and IL-2/JES6 (figure 2H). At very low concentrations (0.01–0.1 ng/mL), both IL-2 and IL-2/JES6 fail to overcome Treg suppressive activity. In contrast, higher concentrations (10 ng/mL for IL-2 and 10–100 ng/mL for IL-2/JES6) restored CD8^+^ T cell proliferation and CD25 expression to levels comparable to those observed in activated CD8^+^ T cells cultured alone. These findings indicate that the suppressive activity of Treg cells depends on IL-2 concentration, supporting a mechanism that involves competition between CD8^+^ T cells and Tregs for circulating IL-2. Taken together, these studies show that Treg cells do not suppress activated CD8^+^ T cells when sufficient concentrations of IL-2/JES6 are provided.

### IL-2/JES6 synergizes with ICIs in cancer immunotherapy, and treatment timing is crucial

**Figure 3:**
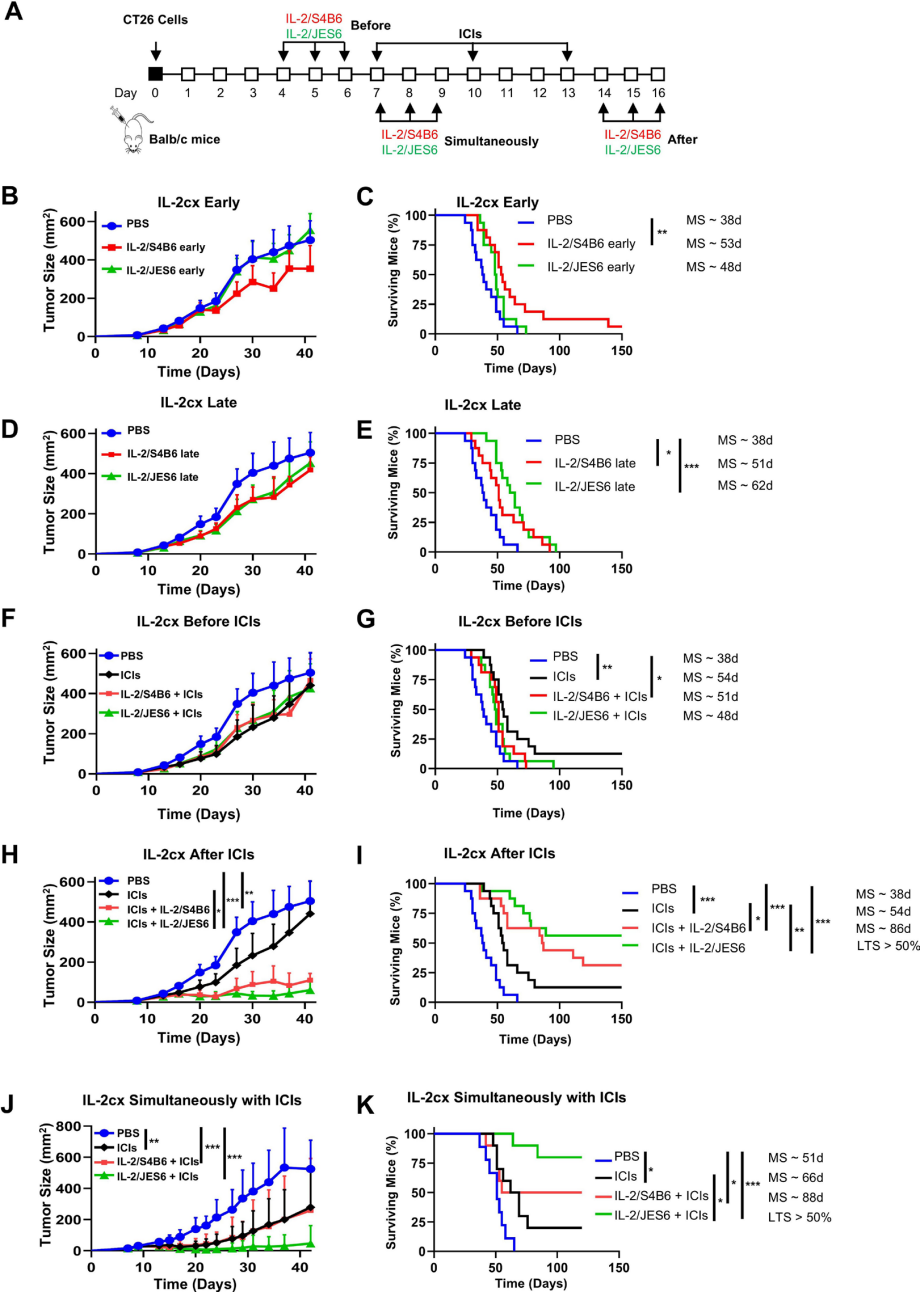
IL-2/JES6 strongly potentiates the antitumor activity of ICIs when administered subsequently. (**A–K**) BALB/c mice were s.c. inoculated with 2×10^5^ CT26 cells on day 0. Mice were i.p. injected with ICIs (αCTLA-4 + αPD-1 antibodies; 0.5 mg/kg each per dose), IL-2cx (2 µg IL-2/dose), or both. Control mice were i.p. injected with the same volume (250 µL) of PBS. A schematic of the study is shown (**A**). IL-2cx was administered on days 4, 5, and 6 (IL-2cx Early) (**B, C**) or on days 14, 15, and 16 (IL-2cx Late) (**D, E**). Alternatively, IL-2cx was administered before treatment with ICIs (**F, G**) or after treatment with ICIs (**H, I**) or within the treatment with ICIs on days 7, 8 and 9 (**J, K**). Tumor growth (**B, D, F, H, J**) and survival of mice (**C, E, G, I, K**) were monitored. Data were pooled from two independent experiments with n=16 for each experimental group except for J and K, which showed one experiment with n=10. Each experimental point represents average±SD. Tumor growth was analyzed by one-way ANOVA, followed by Dunnett’s post hoc test. Survival was analyzed by the Mantle-Cox log-rank test (*p<0.05; **p<0.01; ***p<0.001). Median survival (MS) is shown for each experimental group, except in cases where the proportion of long-term surviving (LTS) mice exceeded 50%. αCTLA-4, anti-cytotoxic T-lymphocyte-associated antigen 4; ANOVA, analysis of variance; αPD-1, anti-programmed cell death 1; ICIs, immune checkpoint inhibitors; IL-2, interleukin-2; IL-2cx, anti-IL-2 antibodies; i.p., intraperitoneally; PBS, phosphate-buffered saline; s.c., subcutaneously.

We decided to investigate the antitumor activity of IL-2cx (2 µg IL-2/dose) either alone or in combination with ICIs (anti-CTLA-4 plus anti-PD-1 antibodies) using the CT26 mouse colon carcinoma model (figure 3A). We first evaluated the antitumor activity of IL-2cx treatment alone, administered either early (days 4, 5 and 6) or late (days 14, 15 and 16). We found that, in both treatment schedules, neither IL-2cx significantly inhibited tumor growth (figure 3B and D). However, in the early treatment schedule, IL-2/S4B6 significantly prolonged median survival of CT26 tumor-bearing mice (by ∼40%) compared with control treatment and completely cured 1 out of 16 mice (figure 3C). In the late treatment schedule, both IL-2/S4B6 and IL-2/JES6 significantly prolonged survival (by ∼34% and ∼63%, respectively) relative to control mice (figure 3E). Notably, the CD25-biased IL-2/JES6, which is typically applied to achieve immunosuppression, shows surprisingly high antitumor efficacy under these conditions. None of the IL-2cx treatment regimens led to body weight reduction, indicating that there were no obvious signs of toxicity (online supplemental figure 4A and B).

We then explored potential synergy between IL-2cx and ICIs and investigated whether the order of treatment administration would impact disease progression. The CT26 tumor model is known to be immunogenic and therefore responsive to ICI treatment.^30^ To partially, but not fully, control tumor growth in this model, each ICI antibody was dosed at 0.5 mg/kg on days 7, 10 and 13 post-tumor cell inoculation for all experiments. Using this treatment schedule, ICIs alone did not significantly inhibit tumor growth (figure 3F) but prolonged the survival of tumor-bearing mice by ∼42% compared with control mice and completely cured 2 out of 16 mice (figure 3G). Administration of either IL-2/S4B6 or IL-2/JES6 (2 µg IL-2/dose) before ICIs did not affect tumor growth compared with ICIs alone (figure 3F) and led to slightly worse (though not statistically significantly) survival (figure 3G). In sharp contrast, administration of IL-2cx after ICIs dramatically improved tumor growth inhibition and survival compared with ICI treatment alone (figure 3H and I). Furthermore, IL-2/JES6 complexes were more effective than IL-2/S4B6 in completely curing mice (9 vs 5 out of 16 mice, respectively). Again, none of the treatment regimens led to body weight loss (online supplemental figure 4C and D). Of note, mice completely cured by ICIs plus IL-2cx were resistant to rechallenge with CT26 cells (online supplemental figure 4E and F), suggesting that robust, long-term immune memory was formed during the treatment. Simultaneous administration of ICIs and IL-2cx also led to potent antitumor activity, completely curing 8 out of 10 mice treated with ICIs+IL-2/JES6 and 5 out of 10 mice treated with ICIs+IL-2/S4B6 (figure 3J and K), indicating that simultaneous treatment is at least as potent as treatment with IL-2cx after ICIs (online supplemental figure 4G).

Immune phenotyping studies revealed that administration of IL-2/JES6 (2 µg IL-2/dose) after ICIs (αCTLA-4 + αPD-1 antibodies; 0.5 mg/kg each per dose) led to significant accumulation of CD8^+^ T cells in tumors compared with either ICI or IL-2/JES6 treatment alone (online supplemental figure 5A). Interestingly, IL-2/JES6 treatment alone, but not IL-2/JES6 treatment after ICIs led to increased tumor accumulation of Treg cells (online supplemental figure 5B). Treg cell counts in the spleens of mice treated with IL-2/JES6 alone or IL-2/JES6 after ICIs were comparable (online supplemental figure 5E). We also observed increased expression of the activation/effector markers CD25, NKG2D, CX3CR1 and CD39 on the surface of CD8^+^ T cells in tumor, spleen, and blood following treatment with IL-2/JES6 alone or after ICIs (online supplemental figure 5D-J).

When we compared the phenotype of CD4^+^ and CD8^+^ T cells in the tumors and spleens of mice treated with IL-2/JES6 before and after ICIs, we found that the latter treatment schedule leads to increased expression of PD-1, TIM-3, and LAG-3 (markers of terminally differentiated T cells) as well as CD25, KLRG1, NKG2D, and perforin (markers of effector cells) (online supplemental figure 6). Thus, we conclude that the superior antitumor efficacy of ICIs+IL-2/JES6 treatment is based on production of a robust population of terminally differentiated T cells that exhibit intact effector function. Collectively, these studies demonstrated that administration of IL-2/JES6 after (but not before) ICIs led to robust immune stimulation and powerful antitumor activity.

### IL-2/JES6 treatment shows low toxicity, thus enabling high-dose treatment with superior efficacy

Since a major limitation of IL-2-based therapies is their toxicity,^8^ we compared the toxicity of IL-2/S4B6 and IL-2/JES6, representing CD25-blocking and CD25-biased IL-2cx, respectively. In a first study, we injected BALB/c mice with IL-2cx daily for eight consecutive days with 2 µg IL-2/dose (online supplemental figure 7A). IL-2/S4B6 induced rapid weight loss (online supplemental figure 7C), dramatic drop in body temperature (online supplemental figure 7D), and death of all treated mice after the fourth or fifth dose (online supplemental figure 7E). In contrast, IL-2/JES6 led to only modest reduction in body weight loss (˂4%) (online supplemental figure 7C), did not induce hypothermia (online supplemental figure 7D), and did not lead to death of any mice over the full course of treatment (online supplemental figure 7E).

We then performed a dose escalation study in which BALB/c mice were treated with either IL-2cx daily for three consecutive days using 2, 4 and 6 µg IL-2/dose for IL-2/S4B6 or 8, 12 and 16 µg IL-2/dose for IL-2/JES6 (online supplemental figure 7B). As in the first study, IL-2/S4B6 showed significantly higher toxicity compared with IL-2/JES6 in terms of weight loss, hypothermia, and mortality (online supplemental figure 7F-K). Mice injected with 2 µg IL-2/dose were the only group with no deaths within the IL-2/S4B6 treatment cohorts, whereas IL-2/S4B6 at 4 and 6 µg IL-2/dose caused 25% and 100% mortality, respectively. On the other hand, IL-2/JES6 was only lethal at the highest dose (16 µg), where treatment led to 12.5% mortality. Thus, we concluded that IL-2/JES6 is at least four times less toxic than IL-2/S4B6.

**Figure 4:**
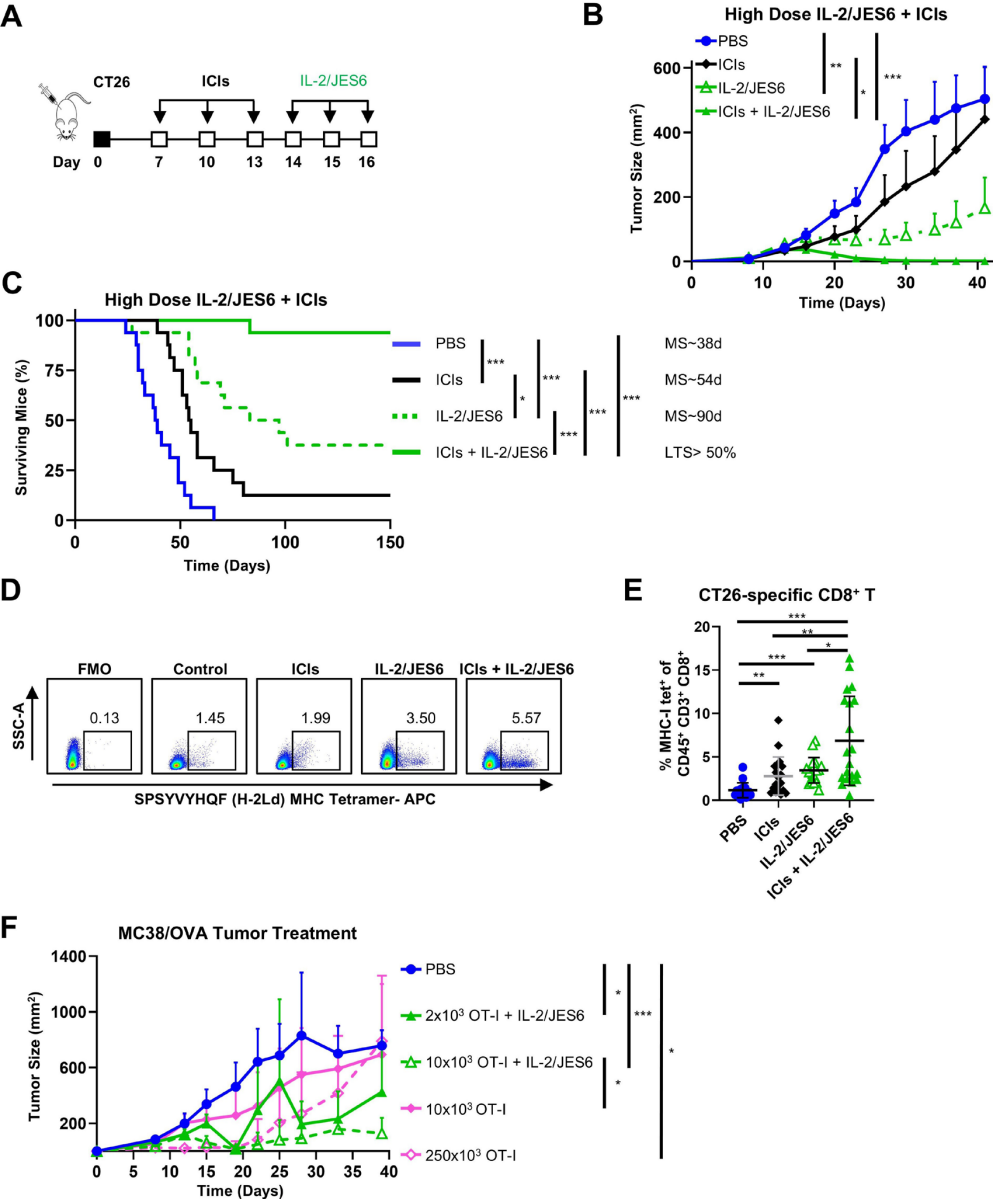
Combination of ICIs with high-dose IL-2/JES6 leads to powerful antitumor activity. (**A–F**) BALB/c mice were s.c. inoculated with 2×10^5^ CT26 cells on day 0. Mice were i.p. injected with ICIs (αCTLA-4 + αPD-1 antibodies; 0.5 mg/kg each per dose), IL-2/JES6 (8 µg IL-2/dose), or both. Control mice were i.p. injected with the same volume (250 µL) of PBS. A schematic of the study is shown (**A**). Tumor growth (**B**) and survival of mice (**C**) were monitored. Data were pooled from two independent experiments with n=16 for each experimental group. (**D–E**), BALB/c mice were inoculated with CT26 cells and treated with ICIs and IL-2/JES6 as in (**A**). Mice were euthanized on day 19. Peripheral blood cells were stained with SPSYVYHQF/H-2L^d^-APC or PE tetramer and analyzed by flow cytometry. Tetramer positive cells (%) in CD8^+^ T cells from one representative mouse (**D**) and with depicted average±SD for all experimental groups (**E**) are presented. FMO: fluorescence minus one control (no tetramer stain). Data were pooled from three independent experiments with n=18–20 for each experimental group. Results were statistically analyzed by unpaired t-test (*p<0.05; **p<0.01; ***p<0.001) (**F**), CD3ε^−/−^ mice were s.c. inoculated with 1×10^6^ MC38/OVA on day 0. Purified CD8^+^ T cells from OT-I /RAG1^−/−^/Ly5.1 mice were adoptively transferred into these mice on day 5. IL-2/JES6 (4 µg IL-2/dose) was i.p. injected on days 6, 7, and 8, and tumor growth was monitored. The experiment was performed twice with comparable results (n=5–6). Each experimental point represents average±SD. Tumor growth was analyzed by one-way ANOVA, followed by Dunnett’s post hoc test. Survival was analyzed by the Mantle-Cox log-rank test (*p<0.05; **p<0.01; ***p<0.001). Median survival (MS) is shown for each experimental group, except in cases where the proportion of long-term surviving (LTS) mice exceeded 50%. αCTLA-4, anti-cytotoxic T-lymphocyte-associated antigen 4; ANOVA, analysis of variance; αPD-1, anti-programmed cell death 1; ICIs, immune checkpoint inhibitors; IL-2, interleukin-2; i.p., intraperitoneally; MHC, major histocompatibility complex; OVA, ovalbumin; PBS, phosphate-buffered saline; s.c., subcutaneously; SSC-A, side scatter-area.

Antitumor activity of IL-2cx in combination with ICIs shown in figure 3 was evaluated using 2 µg IL-2/dose daily for three consecutive days, which is near the maximum tolerated dose of IL-2/S4B6, but well below that of IL-2/JES6. We asked whether the overall antitumor effect of IL-2/JES6 after ICI treatment could be improved by increasing the dosage of IL-2/JES6 to the well-tolerated 8 µg IL-2/dose (online supplemental figure 7K). Indeed, ICI treatment followed by IL-2/JES6 administration potently inhibited tumor growth and completely cured 15 out of 16 experimental mice (figure 4A–C). Interestingly, IL-2/JES6 alone also demonstrated considerable antitumor activity at this dose, significantly inhibiting tumor growth and completely curing 6 out of 16 mice. None of the treatment regimens caused body weight loss (online supplemental figure 8A), confirming the lack of toxicity for IL-2/JES6 at high doses, alone or in combination with ICIs. Mice completely cured by IL-2/JES6 alone or ICIs plus IL-2/JES6 were highly resistant to CT26 tumor rechallenge (online supplemental figure 8B and C), demonstrating that treatment led to robust, durable antitumor immunity.

The immunodominant CD8^+^ T cell response against CT26 tumor cells is directed against a tumor/self-antigen, GP70_423–431_, also known as AH1.^31^ Using AH1-derived SPSYVYHQF/H-2L^d^ tetramers, we determined the relative number of CT26-specific CD8^+^ T cells in the blood of CT26 tumor-bearing mice from the various treatment cohorts in our high-dose study (figure 4A). We found that ICIs plus IL-2/JES6 significantly increased the frequency of CT26-specific CD8^+^ T cells in comparison to treatment with ICIs or IL-2/JES6 alone (figure 4D and E), demonstrating synergy between blockade of T cell inhibitory signals with ICIs and selective stimulation of CD25^+^ T cells with IL-2/JES6. We further analyzed CD62L expression on CT26-specific CD8^+^ T cells and found that CD62L^−^CT26-specific CD8^+^ T cells (ie, effector cells capable of infiltrating non-lymphoid peripheral tissues including the tumor) were the dominantly expanded population in mice treated with ICIs plus IL-2/JES6, with ∼12-times higher abundance of this population compared with control mice (online supplemental figure 8D and F). On the other hand, the relative numbers of CD62L^+^CT26-specific CD8^+^ T cells (ie, naïve cells) were comparable in control (phosphate-buffered saline (PBS)-treated) animals and mice treated with ICIs, IL-2/JES6, and ICIs plus IL-2/JES6 (online supplemental figure 8D and E). To verify that IL-2/JES6 expands tumor-specific CD8^+^ T cells which are subsequently able to reject cancer, we used a model of MC38 colon carcinoma expressing OVA (MC38/OVA)^32^ growing in CD3ε^−/−^ mice with adoptively transferred OT-I CD8^+^ T cells. Indeed, when these mice were treated with IL-2/JES6, the number of OT-I CD8^+^ T cells required to control the growth of MC38/OVA was >100 fold lower than that required in the absence of IL-2/JES6 treatment (figure 4F). Collectively, these analyses showed that ICIs plus IL-2/JES6 treatment leads to effective anti-cancer activity through potent expansion of tumor-specific CD8^+^ T cells that exhibit effector phenotype.

### CD25-biased ICs containing human IL-2 synergize with ICIs to eradicate tumors

To illustrate the translational relevance of our approach, we asked whether the CD25-biased IL-2cx in our ICI combination studies could be replaced with a fully human single-agent cytokine/antibody FP (termed an IC). To this end, we took advantage of a previously designed and characterized panel of CD25-biased ICs comprizing human IL-2 and the anti-human IL-2 antibody F5111, or affinity variants thereof.^17 33^ The F5111 antibody sterically blocks CD122 binding to IL-2 while also allosterically reducing CD25 affinity for IL-2, and CD25 engagement by the IC is therefore required to induce antibody dissociation from the cytokine and enable signaling.^33^ ICs were formulated as previously described, with the C-terminus of IL-2 linked to the N-terminus of the F5111 antibody light chain via a flexible 35 amino acid linker (online supplemental figure 9A).^17^ We used four distinct ICs: one containing the parental F5111 antibody (denoted F5111 IC); one containing the F5111.2 antibody, which was engineered to have ≈16-fold higher affinity for IL-2 compared with the parental clone^33^ (denoted F5111.2 IC); one containing the F5111 variant antibody Y33, which contains a single-point alanine mutation in the light chain variable region that lowers the affinity for IL-2 compared with the parental clone (denoted Y33 IC)^17^; and one containing an irrelevant isotype-matched anti-fluorescein isothiocyanate (FITC) antibody, denoted Control IC. Together, these molecules cover a range of intramolecular affinities between the cytokine and antibody within the IC (in order from weakest to strongest: Control IC; Y33 IC; F5111 IC; F5111.2 IC), thus varying in their extent of CD25 bias.

**Figure 5:**
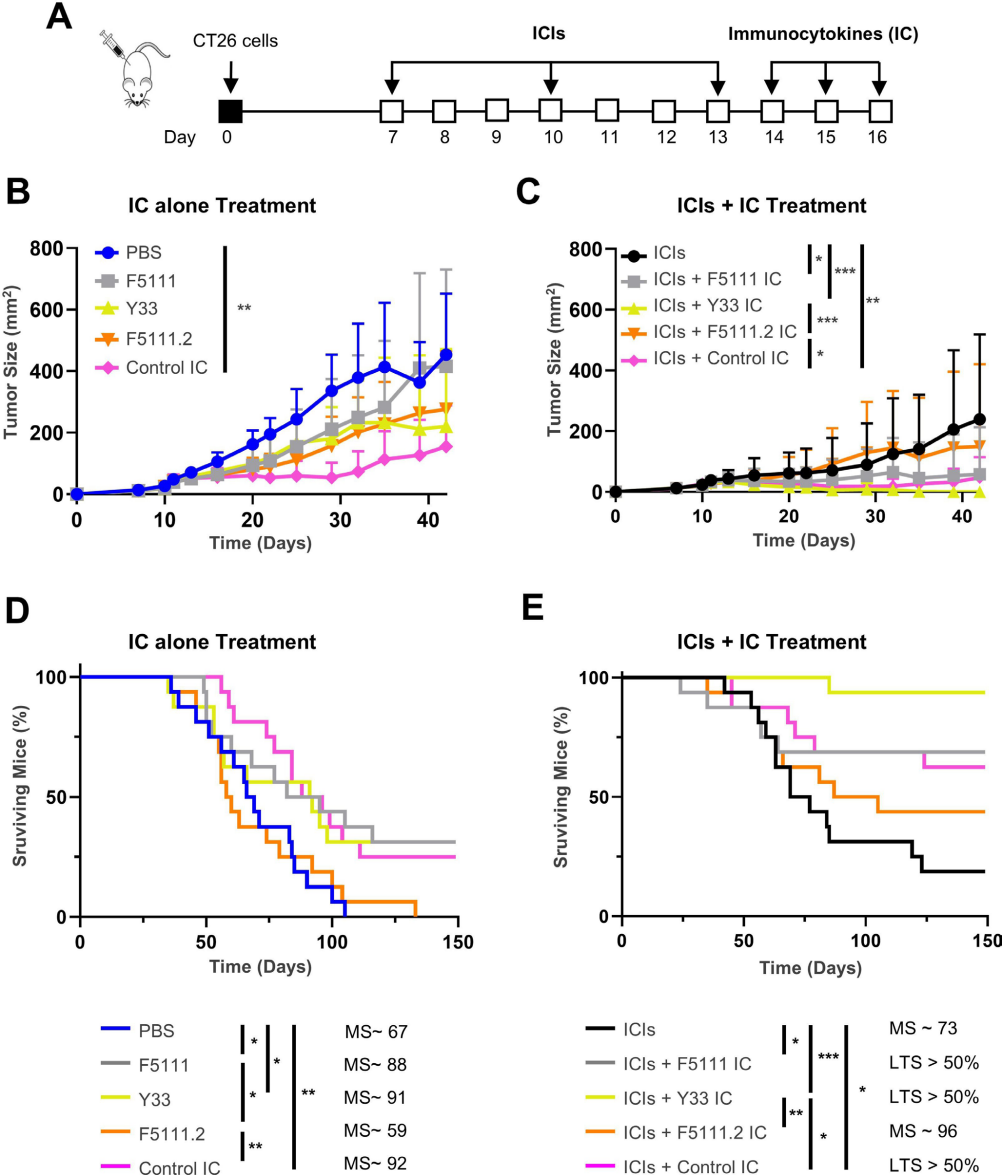
CD25-biased single-chain fusion protein Y33 IC potentiates the antitumor activity of ICIs. (**A–E**) BALB/c mice were s.c. inoculated with 2×10^5^ CT26 cells on day 0. Mice were i.p. injected with ICIs (αCTLA-4 + αPD-1 antibodies; 0.5 mg/kg each per dose), hIL-2-based ICs (2 µg IL-2/dose), or combined ICIs+IC therapies. Control mice were i.p. injected with the same volume (250 µL) of PBS. A schematic of the study is shown (**A**) Tumor growth (**B, C**) and survival of mice (**D, E**) were monitored. Data are shown from one to two independent experiments with n=8 for tumor growth, and data were pooled from two independent experiments with n=16 for survival. Each experimental point represents average±SD. Tumor growth was analyzed by one-way ANOVA, followed by Dunnett’s post hoc test. Survival was analyzed by the Mantle-Cox log-rank test (*p<0.05; **p<0.01; ***p<0.001). Median survival (MS) is shown for each experimental group, except in cases where the proportion of long-term surviving (LTS) mice exceeded 50%. αCTLA-4, anti-cytotoxic T-lymphocyte-associated antigen 4; ANOVA, analysis of variance; αPD-1, anti-programmed cell death 1; ICIs, immune checkpoint inhibitors; i.p., intraperitoneally; s.c., subcutaneously.

We assessed the antitumor activity of our panel of ICs alone and in combination with ICIs in the CT26 mouse model, using the previously established dosing schedule with 2 µg IL-2/dose (figure 5A). The control IC was the only IC that significantly inhibited tumor growth in this model (figure 5B). However, all ICs except for F5111.2 ICs significantly prolonged survival to a similar extent (31–37% compared with control PBS-treated mice) and completely cured 4–5 out of 16 mice (figure 5D). Administration of ICs after ICIs led to a powerful antitumor effect. Y33 IC was particularly effective in combination with ICIs, as it almost fully inhibited tumor growth (figure 5C), and led to complete cures in 15 out of 16 mice (figure 5E). The parental F5111 IC and Control IC combined with ICIs completely cured 11 and 10 out of 16 mice, respectively. Interestingly, F5111.2 IC completely cured more mice than ICIs alone (7 vs 3 out of 16 mice) (figure 5E), despite the fact that F5111.2 IC alone showed no antitumor activity (figure 5D). None of the treatments showed evidence of toxicity as determined by mouse weight (online supplemental figure 9B and C). As in experiments with IL-2cx, mice completely cured by ICIs plus ICs or by ICs alone were fully resistant to tumor progression on rechallenge with CT26 cells (online supplemental figure 9D–G), confirming the durability of antitumor immunity elicited by these treatments.

To corroborate our findings in the CT26 model, we tested the antitumor potential of Y33 IC alone and combined with ICIs in the MC38 colon carcinoma model (online supplemental figure 10A). Again, ICIs and Y33 IC demonstrated strong synergy, almost fully inhibiting MC38 tumor growth and completely curing five out of eight mice (online supplemental figure 10B and C). As in the CT26 model, no weight loss was observed in any of the treatment cohorts (online supplemental figure 10D). We also interrogated the role of CD4^+^ and CD8^+^ T cells in the antitumor activity of ICIs plus Y33 IC in CT26 tumor-bearing mice (online supplemental figure 11A). Simultaneous depletion of CD4^+^ and CD8^+^ T cells completely abrogated the therapeutic effect of ICIs plus Y33 IC, thus demonstrating that T cells are the predominant immune cells responsible for tumor rejection (online supplemental figure 11B and C). Moreover, depletion of CD8^+^ T cells only dampened antitumor activity to considerably higher extents than depletion of CD4^+^ T cells only, establishing that the dominant immune population mediating the observed therapeutic effect of ICIs plus Y33 IC was CD8^+^ T cells. Once again, no weight loss was observed in any of the treatment cohorts (online supplemental figure 11D).

**Figure 6:**
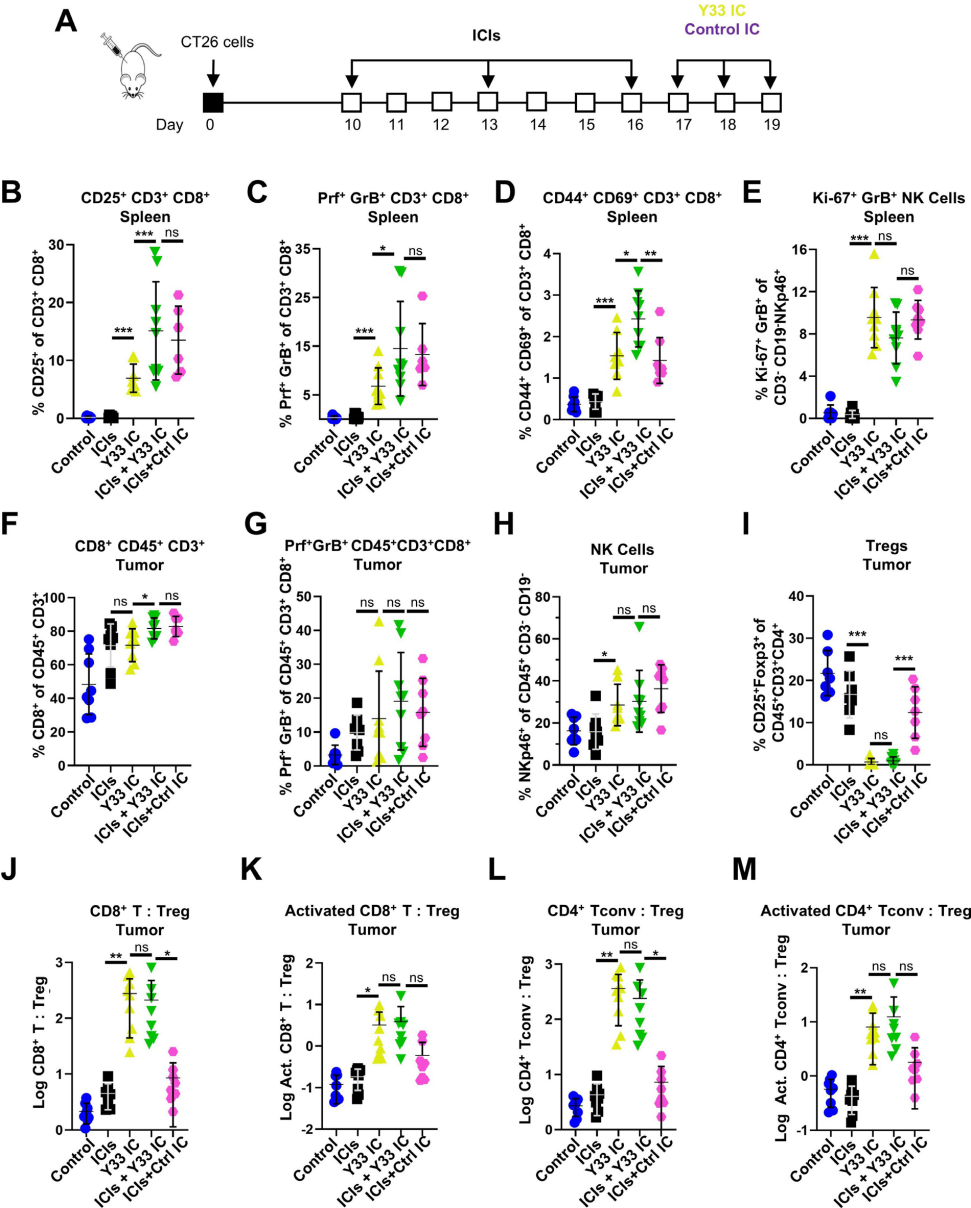
Combining the Y33 IC with ICIs leads to robust activation of effector T cell immunity in CT26 tumor-bearing mice. (**A–M**) BALB/c mice were s.c. inoculated with 2×10^5^ CT26 cells on day 0. Mice were i.p. injected with ICIs (αCTLA-4 + αPD-1 antibodies; 0.5 mg/kg each per dose), hIL-2-based ICs (2 µg IL-2/dose), or combined ICIs+IC therapies. Control mice were i.p. injected with the same volume (250 µL) of PBS. A schematic of the study is shown (**A**). Flow cytometry analysis of various lymphocyte populations in the spleen (**B–E**) and in the tumor (**F–M**) was performed on day 21. Average±SD for each experimental group is shown. Each point represents an individual mouse. Data were pooled from two independent experiments with n=6–9 for each experimental group. Activated CD8^+^ T cells gated as CD3^+^CD8^+^CD25^+^Foxp3^−^ cells; activated CD4^+^ T_conv_ cells gated as CD3^+^CD4^+^CD25^+^Foxp3^−^ cells. Results were statistically analyzed by unpaired t-test (*p<0.05; **p<0.01; ***p<0.001). αCTLA-4, anti-cytotoxic T-lymphocyte-associated antigen 4; αPD-1, anti-programmed cell death 1; ICs, immunocytokines; ICIs, immune checkpoint inhibitors; i.p., intraperitoneally; NK, natural killer; ns, not significant; PBS, phosphate-buffered saline; s.c., subcutaneously; T_conv_, conventional T cells; Tregs, regulatory T cells.

To analyze the mechanistic correlates of disease control in the context of ICI plus IC combination therapies, we quantified activation and effector markers within CD8^+^ T cells as well as Treg abundance in CT26 tumor-bearing mice treated with ICIs alone, Y33 IC alone, and ICIs plus either Y33 IC or Control IC (figure 6A). Whereas ICIs alone did not affect the relative number of CD25^+^ and Prf^+^GrB^+^CD8^+^ T cells in the spleen, Y33 IC alone and, to a greater extent, ICIs plus ICs, increased the frequency of both populations (figure 6B and C). Furthermore, the ICIs plus Y33IC treatment group was more efficient than all other cohorts in inducing CD44^+^CD69^+^CD8^+^ T cells in the spleen (figure 6D). Y33 IC alone and ICIs plus IC increased levels of Ki-67^+^GrB^+^ NK cells in the spleen, whereas ICIs alone did not (figure 6E). Within the tumor microenvironment, ICIs alone, Y33 IC alone, and ICIs plus ICs increased relative counts of total CD8^+^ T and Prf^+^GrB^+^CD8^+^ T cells, with ICIs plus ICs inducing significantly higher relative counts (figure 6F and G). Y33 IC alone and ICIs plus ICs increased levels of NK cells in the tumor, whereas ICIs alone did not (figure 6H). Strikingly, whereas ICIs alone and ICIs plus Control IC did not appreciably affect tumor Treg cell counts compared with untreated mice, Y33 IC alone and ICIs plus Y33IC significantly lowered relative counts of Treg cells in the tumor microenvironment (figure 6I). Consequently, the ratios of both total and activated CD8^+^ and CD4^+^ T cells relative to Treg cells in the tumor more strongly favored the effector cells following Y33 IC alone or ICIs plus Y33 IC treatment compared with untreated mice and mice treated with ICIs alone or ICIs plus Control IC (figure 6J–M). Overall, in vivo studies demonstrated the potent antitumor effects of the CD25-biased Y33 IC across multiple animal models, due in large part to the activities of CD8^+^ T cells.

### Y33 IC stimulates expansion and effector molecule expression in antigen-primed CD8^+^ T cells

**Figure 7:**
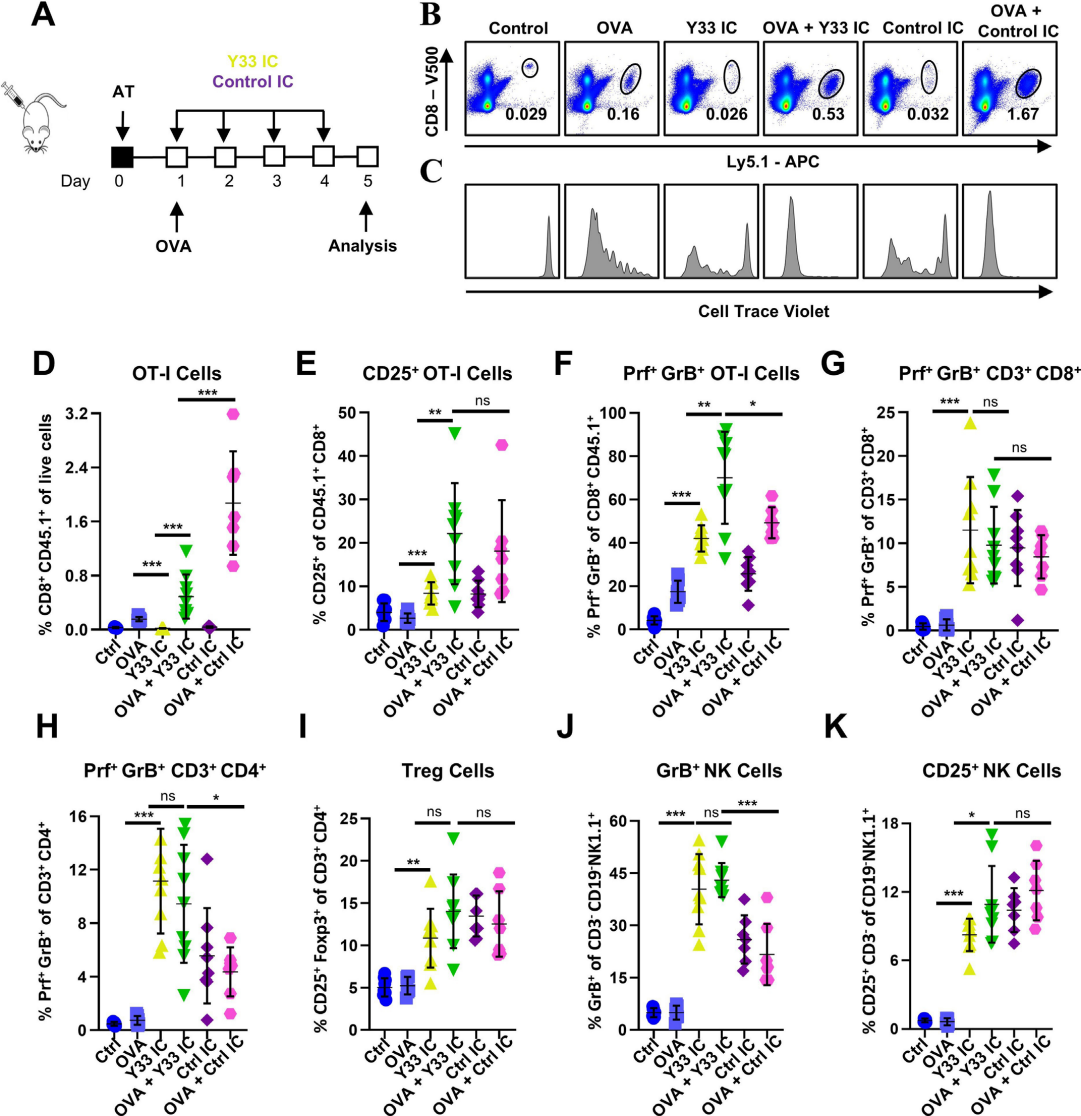
Y33 IC expands antigen-primed CD8^+^ T cells and significantly boosts their expression of effector molecules. (**A–K**) Purified CD8^+^ T cells from OT-I/RAG1^−/−^/Ly5.1 mice were adoptively transferred (AT) into B6 mice. Mice were i.p. injected with 350 µg OVA, hIL-2-based ICs (2 µg IL-2/dose), or OVA+ICs, and their spleens were analyzed by flow cytometry. A schematic of the study is shown (**A**), as are dot plots showing the expansion of AT CD8^+^ T cells in one representative mouse (**B**) and their respective CTV profile (**C**). Flow cytometry analysis of various lymphocyte populations in the spleen is presented (**D–K**). Average±SD for each experimental group is shown. Each point represents an individual mouse. Data were pooled from two independent experiments with n=6–9 for each experimental group. Results were statistically analyzed by unpaired t-test (*p<0.05; **p<0.01; ***p<0.001). CTV, CellTrace Violet; IC, immunocytokine; i.p., intraperitoneally; NK, natural killer; ns, not significant; OVA, ovalbumin; Treg, regulatory T cell.

To further dissect the mechanistic basis for Y33 IC-induced CD8^+^ T cell potentiation in mouse tumor models, we evaluated the potential of this IC to stimulate expansion and effector molecule expression in antigen-primed CD8^+^ T cells. To this end, we employed an adoptive transfer model in which congenic healthy mice with transferred OT-I CD8^+^ T cells were injected with OVA or PBS and subsequently treated with Y33 IC or Control IC (figure 7A). Spleens were analyzed by flow cytometry 24 hours after the final treatment. In OVA-experienced mice, both Y33 IC and Control IC induced proliferation and expansion of adoptively transferred cells, with Control IC showing a more pronounced effect than Y33 IC (figure 7B–D). Expansion by both ICs was found to be antigen-specific, as no expansion was observed in the absence of OVA priming. Moreover, Y33 IC and Control IC induced strong expression of CD25, perforin, and granzyme B in antigen-primed CD8^+^ T cells (figure 7E and F). ICs also increased expression of perforin and granzyme B in endogenous polyclonal CD8^+^ and CD4^+^ T cells in the presence or absence of OVA priming (figure 7G and H). Y33 IC and Control IC expanded Treg cells to a similar extent (figure 7I). Additionally, Y33 IC induced more GrB^+^ NK cells compared with Control IC (figure 7J), although both treatments led to similar frequencies of CD25^+^ NK cells (figure 7K). All of the above analyses were performed using relative cell counts. We also reanalyzed this data with respect to absolute cell numbers (online supplemental figure 12). The overall trends remained consistent with those in figure 7. We note that the experimental groups treated with control IC showed a somewhat greater increase in spleen cellularity than those treated with Y33 IC. This difference is likely due to the unbiased activity of IL-2 within control IC, which broadly stimulates all IL-2-responsive cells, particularly Tregs and NK cells.

As adoptive transfer studies showed that Control IC potentiates antigen-primed CD8^+^ T cells to a similar extent as Y33 IC, we sought to determine whether Y33 IC would show an advantage in terms of toxicity, due to its CD25 bias. We determined the extent of pulmonary edema, a frequent toxicity of clinically dosed IL-2,^7^ induced by treatment with Y33 IC compared with IL-2/JES6 and Control IC for 3 consecutive days at 2 µg IL-2/dose, a schedule that was found to be therapeutic in our tumor models (online supplemental figure 13A). Whereas IL-2/JES6 and Control IC treatment led to significant retention of water in the lungs compared with PBS-treated control mice, demonstrative of lung edema, the single-chain CD25-biased Y33 IC did not (online supplemental figure 13B). Furthermore, on injection of mice with 2 µg IL-2/dose Y33 IC or Control IC for 8 consecutive days (online supplemental figure 13C), significant body weight loss and hypothermia were observed in mice treated with Control IC but not with the CD25-biased Y33 IC (online supplemental figure 13 D and E). Moreover, IL-2/JES6 and Y33 IC treatments did not cause a significant increase in serum levels of cytokines associated with cytokine release syndrome, such as interferon-gamma (IFN-γ) and IL-6, indicating a low toxicity profile. In contrast, Control IC treatment significantly increased serum levels of IFN-γ, IL-10, CXCL-9, CXCL-10, TNFα, IL-6, and CCL2, indicating a potential risk of toxicity (online supplemental figure 14). These data indicate that CD25-biased IL-2 therapies show superior safety profiles compared with therapies based on the natural IL-2 cytokine. Collectively, these studies reveal that Y33 IC potently stimulates CD8^+^ T cell expansion, activation, and effector functions without eliciting toxicities typically associated with IL-2 treatment, rendering this molecule a promising candidate for cancer immunotherapy.

## Discussion

This study provides several important conceptual and translational advancements in the field of IL-2-based cancer immunotherapy. Notably, clinical trials using engineered IL-2 variants designed to preferentially stimulate intermediate-affinity IL-2R (ie, CD25-blocking agonists) have yielded disappointing outcomes in patients with cancer.^28^ These findings underscore the need to re-evaluate IL-2 biology and explore alternative drug design strategies to improve therapeutic efficacy. Recent studies in models of chronic viral infection and cancer have demonstrated that targeting intermediate-affinity IL-2Rs is insufficient to elicit robust antiviral or antitumor responses. In contrast, engagement of the high-affinity trimeric IL-2R, particularly in combination with PD-1 blockade, has been shown to induce potent therapeutic effects, highlighting the importance of selectively activating cells with high levels of CD25 for successful immunotherapy.^22 23 34^ However, selective targeting of CD25 on tumor-specific T cells, which transiently upregulate this receptor on antigen recognition, remains a major challenge, as CD25-biased IL-2 agonists inevitably also stimulate Tregs, which constitutively express high levels of CD25. This results in their robust expansion and functional enhancement, thereby promoting immunosuppression and potentially antagonizing antitumor immunity.^2 14 33^ Accordingly, CD25-biased IL-2 agonists have been predominantly used to expand Tregs in settings of autoimmunity and transplantation.^28 35^ Designing IL-2 variants that can engage CD25 on tumor-specific T cells while minimizing Treg activation remains a central goal in engineering this cytokine.

Notably, a recent effort to exploit CD25-biased IL-2 signaling in cancer immunotherapy involved the development of an IL-2/CD25 FP, which forms inactive head-to-tail transdimers that slowly dissociate into monomeric, biologically active units.^36^ These FPs exhibit a prolonged half-life (∼16 hour) compared with IL-2 (∼5 min).^37^ Moreover, IL-2/CD25 FP preferentially stimulates Tregs at low doses but also activates effector T cells expressing the high-affinity IL-2R at higher concentrations. Importantly, in the context of cancer immunotherapy, IL-2/CD25 FP was found to amplify vaccine-induced, neoantigen-specific CD4^+^ and CD8^+^ T cell responses, thereby enhancing antitumor immunity.^11^ Subsequent work further demonstrated that high-dose IL-2/CD25 FP supports potent antitumor responses both as a monotherapy and, more effectively, in combination with PD-1 blockade.^21^ These antitumor effects were associated with an increased CD8^+^ T cell:Treg ratio in the tumor microenvironment, expansion and augmented function of tumor-specific CD8^+^ T cells, and a reduction in T cell exhaustion. This treatment also induced durable memory responses that protected against tumor re-emergence,^21^ illustrating the therapeutic potential of IL-2 agonists that selectively target CD25 on antigen-experienced effector T cells in the context of checkpoint inhibition for cancer immunotherapy.

Our study employed an alternative strategy towards biasing IL-2 based on complexing or fusing IL-2 with anti-IL-2 antibodies. We explored the relative merits of employing CD25-biased versus CD25-blocking anti-IL-2 antibodies, revealing the unexpected therapeutic advantage for CD25-biased anti-IL-2 antibodies in the context of ICI combination therapy for cancer treatment. CD25-biased IL-2cx undergoes a distinct mechanism of action compared with IL-2/CD25 FP, where the JES6-1 antibody sterically blocks engagement of the CD122 and CD132 receptor subunits and allosterically exchanges with CD25 for cytokine engagement.^14 18 38^ Thus, the dynamics of IL-2 availability differ between IL-2/CD25 FP, which requires spontaneous transdimer dissociation, and IL-2cx, and our work clarifies the impact of these unique mechanistic activities. Moreover, IL-2cx and ICs deliver IL-2 together with an antibody, which results in pharmacological advantages such as extended serum half-life due to neonatal Fc receptor-mediated recycling.^39^ Furthermore, this study gained novel insight into the importance of administration timing when combining IL-2cx and ICs with ICIs. We hypothesized that administering CD25-biased IL-2cx or ICs in an immunologically favorable context, such as following immune checkpoint blockade to reinvigorate immunosuppressed effector T cells in the tumor microenvironment, could preferentially activate tumor-specific effector T cells. Our findings supported this concept, providing important mechanistic insights relevant to cytokine-based drug design and showcasing the previously underestimated potential of CD25-biased IL-2cx and ICs in cancer immunotherapy, particularly in combination with ICIs.

Here we show that CD25-biased IL-2cx or ICs, when administered in combination with ICIs, showed powerful antitumor activity, despite inducing robust expansion of Treg cells. The strong anticancer activity of CD25-biased IL-2cx reflects their superior potential to stimulate recently activated CD8^+^ T cells (on which CD25 is upregulated), which overcomes Treg-mediated suppression of CD8^+^ T cells. IL-2 sequestration by Treg cells was shown to be a key mechanism for CD8^+^ but not CD4^+^ T cell suppression.^40^ Since CD25-high Treg cells compete with activated CD8^+^ T cells for the limited amount of IL-2 produced by the CD8^+^ T cells, the sequestration mechanism is very efficient in this context.^41 42^ However, administration of high-dose IL-2cx with extended half-life^14 15^ leads to supersaturating concentrations of the cytokine, providing strong and long-term IL-2R triggering in activated CD8^+^ T cells, even when increased counts of Treg cells are present. This mechanistic decoupling of Treg expansion from functional suppression is a key insight of our work and underpins the therapeutic efficacy of CD25-biased IL-2 agonists in the context of immune checkpoint blockade. Another interesting observation in our work was the variability in tumor Treg expansion depending on the IL-2 agonists that were employed. For instance, murine IL-2/JES6 complexes led to significant expansion of Tregs in the tumor (online supplemental figure 5B and E) whereas the human IL-2-based Y33 IC did not (figure 6I). Similar context-dependent behavior of CD25-biased IL-2 agonists was described in a recent study,^22^ which reported that Tregs within the tumor microenvironment are present at high baseline frequencies, and many exist in an activated, proliferative state due to local IL-2 and other biochemical cues. This background activation leaves limited potential for further expansion following exogenous IL-2 delivery. In contrast, Tregs in peripheral compartments (which are mostly in a resting state) respond readily to IL-2 stimulation. Furthermore, whereas peripheral CD8^+^ T cells express little to no CD25, tumor-infiltrating CD8^+^ T cells greatly upregulate CD25 on antigen recognition, enabling them to effectively compete with Tregs for IL-2. Collectively, these findings illustrate how the local immune context shapes the cellular responsiveness to IL-2-based therapies and help explain the context dependence of Treg dynamics in the tumor that was observed in our study.

While these mechanisms explain how CD25-biased IL-2cx can selectively enhance CD8^+^ T cell responses despite Treg expansion, it is important to consider interspecies differences in T cell biology that may influence the translational relevance of these findings. Although human IL-2 is known to be cross-reactive in mice, receptor affinities differ between the species.^14^ Furthermore, in murine models, Tregs typically exhibit uniformly high CD25 expression,^43^ whereas human Tregs display greater heterogeneity in CD25 levels.^44^ Notably, peripheral blood from humans may contain up to 30% CD4^+^ CD25^+^ T cells (compared with 10% in mice), with only approximately 1–2% of cells that exhibit the highest CD25 expression being functionally suppressive (bona fide Tregs).^44^ This abundance of CD25 on human CD4^+^ T cells may limit the availability of CD25-biased IL-2cx or ICs for CD8^+^ effector T cells, highlighting a potential challenge when translating these therapies to clinical settings. Given this interspecies variability, careful dose optimization and patient stratification will be necessary to ensure that CD25-biased IL-2-based immunotherapeutic strategies achieve maximal efficacy in humans.

In addition to interspecies differences, another consideration is whether factors beyond Treg suppression could contribute to the observed effects of CD25-biased IL-2cx in vivo. The observation that broad CD4^+^ T cell depletion allows for robust expansion of adoptively transferred OT-I CD8^+^ T cells strongly suggests that Tregs play a dominant role in suppressing activated T cells; however, additional mechanisms, such as altered availability of homeostatic cytokines (IL-7 and IL-15)^45^ and the loss of CD4^+^ helper T cell support,^46^ could also contribute to this finding. Despite these possibilities, our data indicate that the observed effect is primarily due to Treg depletion. First, CD25-biased IL-2cx and ICs selectively enhance CD8^+^ T cell proliferation and effector function in the presence of Tregs, strongly suggesting that overcoming Treg-mediated suppression is the dominant mechanism. Furthermore, if increased IL-7 and IL-15 availability were driving CD8^+^ T cell expansion, we would expect a broader effect beyond just the adoptively transferred OT-I cells, which was not observed. In addition, others have observed that Treg depletion significantly enhances CD8^+^ T cell responses. While we cannot fully exclude additional mechanisms, our findings strongly support the conclusion that CD25-biased IL-2cx and ICs counteract Treg-mediated suppression of CD8^+^ T cell expansion.

Importantly, we reveal that the timing of IL-2 agonist administration relative to ICI therapy is critical to therapeutic outcome. This finding was not investigated in earlier studies and provides critical translational insight into how these agents can be optimally integrated into clinical regimens. Our study raises an important question as to why there is such a striking difference in antitumor activity when CD25-biased IL-2cx/ICs are administered before (IL-2cx/ICs+ICIs) versus after (ICIs+IL-2cx/ICs) ICIs. Expression of T cell-inhibitory receptors including PD-1 is upregulated after chronic tumor antigen exposure, causing T cell receptor signal unresponsiveness and inability to produce IL-2.^47^ It has been demonstrated that PD-1 blockade reinvigorates IL-2 production in T cells,^22^ and this is an important mechanism for the effectiveness of cancer immunotherapies, as IL-2 neutralization or CD25 blockage strongly diminishes the antitumor efficacy of PD-1 blockade.^22^ Further, IL-2 signaling generates a positive feedback loop by increasing CD25 expression in T cells.^48^ The timing of IL-2 delivery is crucial, as PD-1 signaling upregulates MARCH5, leading to sustained degradation of the γ_c_ and impairing IL-2 signaling in T cells. However, PD-1 blockade restores surface expression of γ_c_, allowing for recovery of IL-2 responsiveness.^49^ Administering IL-2cx after (rather than before) ICIs thus ensures that tumor-specific T cells respond to IL-2cx, thereby maximizing their proliferative and cytotoxic potential. Our studies suggest that IL-2cx/ICs+ICIs treatment leads to limited stimulation of tumor-specific CD8^+^ T cells since T cells in the tumor microenvironment express PD-1 and are exhausted, with little or no CD25 expression. Moreover, concomitant Treg cell expansion induced by CD25-biased IL-2cx/IC may lead to suppression of tumor-specific CD8^+^ T cells, particularly once the concentration of IL-2cx/IC decreases and the CD8^+^ T cells cannot effectively compete for circulating IL-2. Indeed, elevated Treg cell counts remain for >1 week after CD25-biased IL-2cx treatment,^50^ so a scenario of subsaturating IL-2 is likely. It was also shown that IL-2 signaling increases PD-1 expression on Treg cells^51^ and that PD-1 blockade augments the suppressive potential of Treg cells.^51^ Thus, the IL-2cx/IC+ICIs schedule may both induce Treg expansion and amplify Treg suppressive activity. Similarly, CTLA-4 expression in Treg cells is also elevated following treatment with IL-2cx^20^ which may inhibit priming of tumor-specific T cells. Thus, combining IL-2 therapy with CTLA-4 blockade, which enables costimulation in tumor-specific T cells,^27^ would be most effective when checkpoint inhibition is implemented first, allowing for more effective CTLA-4 blockade as well as more robust IL-2-mediated activation due to increased CD25 expression together with elimination of PD-1 and CTLA-4 inhibitory signals on tumor-specific effector T cells. In other words, in the ICIs+IL-2cx/IC schedule, the ICIs serve to unleash tumor-specific T cells to enable the stimulatory activity of IL-2cx/IC. Consistent with our findings, Hashimoto *et al* found that delaying ICIs treatment relative to IL-2 treatment in a model of chronic infection led to higher viral titer and was associated with immunopathology.^23^

One of the limitations of this study is that the employed CT26 and MC38 colon carcinoma models both represent immunogenic tumors with high tumor mutational burden (TMB).^30^ ICIs demonstrate highest efficacy in patients with high TMB tumors such as melanoma or non-small cell lung cancer,^52^ which also have higher numbers of tumor-specific T cells.^52^ Since the strong antitumor activity of ICIs+IL-2cx relies on stimulation of tumor-specific CD8^+^ T cells that are reinvigorated by ICIs, it is possible that efficacy would be lower in poorly immunogenic tumors. To explore this, we extended our analysis to two poorly immunogenic tumor models, 4T1 and LLC (also called LL2), which are well known for their resistance to ICI monotherapy. In both models, the combination of ICIs with CD25-biased IL-2cx showed only limited efficacy (data not shown). These findings are consistent with previous work showing limited benefit of ICIs in non-immunogenic models^52^ and further support the notion that a pre-existing tumor-specific T cell response is crucial for the effectiveness of IL-2-based immunotherapy in combination with ICI. In these cases, we hypothesize that the use of a neoantigen vaccine^11^ to increase the frequency of tumor-specific T cells could be particularly effective in combination with CD25-biased IL-2cx/IC to efficiently stimulate antitumor immunity.

Some previous reports had favored CD25-blocking over CD25-biased IL-2cx in the context of cancer immunotherapy due to CD25 expression on CD31^+^ pulmonary endothelial cells, which can lead to inflammatory immune activation in lung tissue and consequent edema.^53^ However, we demonstrated here that the toxicity of CD25-biased IL-2cx is much lower than that of CD25-blocking IL-2cx. Our study provides a systematic comparison between CD25-biased and CD25-blocking IL-2cx in the context of ICI therapy, demonstrating that CD25-biased IL-2cx display superior therapeutic features, including lower systemic toxicity, more selective activation of tumor-specific T cells, and more robust antitumor activity. This enables the use of high doses for CD25-biased IL-2cx, which can strengthen antitumor activity, as observed in our studies. Higher toxicity of CD25-blocking IL-2cx most likely reflects their much lower selectivity leading to stimulation of a broader spectrum of immune cells, including NK cells^14 15^ and leading to massive production of effector cytokines. Moynihan *et al*^54^ provided key insights into the differential toxicity and efficacy of IL-2 variants that either avoid or engage CD25, further supporting our conclusions regarding the superior therapeutic window of CD25-biased versus CD25-blocking IL-2cx. Their study demonstrated that IL-2 variants designed to avoid CD25 preferentially expand NK cells due to their elevated expression of IL-2Rβ, stimulating high levels of IFN-γ production and leading to systemic toxicity.^54^ These findings align with our results, demonstrating that CD25-biased IL-2cx and ICs can achieve strong antitumor responses with a more favorable safety profile by selectively stimulating recently activated CD8^+^ T cells in the tumor microenvironment while minimizing harmful systemic inflammatory responses driven by NK cells.^54^ It is possible that CD25-blocking IL-2cx may be more efficient in major histocompatibility complex I low or negative tumors where CD8^+^ T cells are inefficient due to their enhancement of NK activity.^55^ We also showed that IL-2/S4B6 expanded naïve CD8^+^ T cells without antigen-priming, thus confirming the previous report that CD25-blocking IL-2cx differentiate naïve CD8^+^ T cells into protective memory-like cells per se.^56^ Nevertheless, at equivalent doses, we found CD25-biased IL-2cx to be as effective or better than CD25-blocking IL-2cx when administered after ICIs. Here, we initiated treatment when tumors reached an average size of 20–30 mm^2^; future work could explore the impact of tumor size at treatment initiation on disease outcome in the context of IL-2cx and ICI combination therapy.

In conclusion, we have demonstrated that CD25-biased IL-2cx or IC can synergize with ICIs to mediate complete rejection of established tumors via potent and selective stimulation of CD25^+^ tumor-specific CD8^+^ T cells. However, we note that timing is essential to maximize the synergistic activity of this combination therapy. The insights gained from this study will be important for the development of effective next-generation cancer immunotherapy strategies.

## Methods

### Cell lines

CT26 murine colorectal carcinoma and MC38 colon adenocarcinoma cell lines from the American Type Culture Collection (Manassas, Virginia, USA) were used in the experiments. MC38/OVA cells were provided by Ondřej Štěpánek from the Institute of Molecular Genetics of the Czech Academy of Sciences (Prague, Czech Republic) and characterized in a study by Horkova *et al*.^32^ Cells were propagated in Roswell Park Memorial Institute (RPMI)-1640 (Sigma-Aldrich) medium supplemented with 10% heat-inactivated fetal bovine serum (FBS), 2 mM glutamine, 100 U/mL penicillin, 100 µg/mL streptomycin, 1 mM sodium pyruvate, 4.5 g/L of glucose, 10 mM HEPES solution, and 5 mL of non-essential amino acids.

### Mice

The inbred BALB/c (H-2d), C57BL/6 (H-2b), transgenic OT-I/Rag2^−/−^/Ly5.1 and OT-II/Rag1^−/−^/Ly5.1 mice and T cell-deficient CD3ε^−/−^ mice were obtained from the mouse facility of the Institute of Microbiology of the Czech Academy of Sciences (Prague, Czech Republic). All mice used for experiments were 9–15 weeks old. Mice were in the 19–22 g body weight range and had unlimited access to food and water. In all animal work, we strictly followed in line with the institutional guidelines for the care and use of laboratory animals, and all experiments were performed in line with a protocol approved by the Institutional Animal Care and Use Committee of the Czech Academy of Sciences (approved experimental project 9–2022 P and approvals MZE-15929/2023–13143, MZE-15929/2023) and in compliance with local and European guidelines.

### Tumor models

Cells were harvested and single-cell suspensions of 2×10^5^ cells (CT26) or 1×10^6^ cells (MC38, MC38/OVA) in 100 µl of RPMI-1640 medium per mouse were subcutaneously (s.c.) injected into the right flank of either BALB/c female mice (CT26) or B6 female mice (MC38, MC38/OVA). Tumor growth and survival were monitored. Treatments were initiated when the average tumor size reached 20–30 mm^2^. For the tumor treatment experiments, tumor-bearing mice were intraperitoneally (i.p.) injected with 0.5 mg/kg of αCTLA-4 and αPD-1 monoclonal antibody (mAb) in 250 µl of PBS three times every 3rd day according to the protocol. IL-2 complexes (2 or 8 µg IL-2/dose) or human IL-2-based ICs were injected in 250 µl of PBS on 3 consecutive days. One day after the last treatment, mice were sacrificed and spleen, peripheral blood, and/or tumor were harvested.

### Antibodies

Antibodies to CD3ε-V500, CD4-V500, CD8a-V500, B220-V500, CD45-V500, KLRG1-FITC were purchased from BD Pharmingen to be used for flow cytometry. Antibodies to the following antigens were purchased from BioLegend: CD25-PE, CD39-PE, CX3CR1-FITC, NKp46-PerCP-Cy5.5, Perforin-FITC, CD39-PE, CX3CR1-FITC, Tim-3-PerCP-Cy5.5. Antibodies to CD3ε-FITC, CD3ε-PE, CD3ε-ef450, CD3ε-PE-Cy7, CD3ε-AF700, CD4-APC, CD4-ef450, CD8a-PerCP-Cy5.5, CD8a-AF700, CD19-ef450, CD25-APC, CD44-AF700, CD45-PE-Cy7, CD45.1-APC, CD45.1-PerCp-Cy5.5, CD49b(DX5)-FITC, CD49b(DX5)-ef450, CD62L-ef450, NK1.1-PE-Cy7, NKp46-APC, NKG2D-APC, Lag-3-PE-Cy7, Tim-3-PE, PD-1-APC, GranzymeB-PE, GranzymeB-PerCPef710, Foxp3-PE, Ki-67-AF700 were purchased from eBioscience. Antibodies to CD8-FITC (clone KT15) and CTLA-4-FITC were purchased from Invitrogen.

Fc-block (anti-CD16/CD32 antibodies; eBioscience) was used both in surface and intracellular staining. αCD4 (GK1.5), αCD8 (53–6.7), and αCD25 (PC-61.5.3) antibodies were purchased from Bio X Cell (Lebanon, New Hampshire, USA) for depletion. αCTLA-4 (9H10) and αPD-1 (RPM1-14) antibodies were purchased from Bio X Cell for immune checkpoint blockade. αmIL-2 antibodies (S4B6, JES6-1A12) for preparing IL-2 complexes, were obtained from Bio X Cell. CTV dye was purchased from Invitrogen (Paisley, UK)

### IL-2cx and IC preparation

IL-2 complexes were prepared by mixing recombinant mouse IL-2 (Miltenyi Biotec, Germany) with anti-mIL-2 mAb S4B6 or JES6-1A12 (both reagents were in PBS) at molar ratio 2:1. The mix was then incubated at room temperature for 15 min and diluted with PBS to the desired concentration and stored at −20^ο^C. Aliquots were thawed shortly before use. All ICs were expressed and purified as described in VanDyke *et al*.^17^

### In vitro proliferation assays

Purified CD4^+^ T and CD8^+^ T cells were plated in flat-bottom plates precoated with anti-CD3 mAb (10 µg/mL) at a volume of 0.2 mL and a density of 5×10^4^ cells/mL. The cells were cultured with titrated amounts of IL-2, IL-2/S4B6 or IL-2/JES6 in the presence or absence of anti-CD25 mAb (10 µg/mL). For Treg suppression assays, magnetic-activated cell sorting (MACS)-purified and, where indicated, CTV-labeled CD8^+^ T cells (5×10⁴ cells/well) were co-cultured with fluorescence-activated cell sorting (FACS)-purified Treg cells at varying ratios in 96-well plates precoated with anti-CD3 mAb (2 µg/mL). IL-2 or IL-2/JES6 (10 ng IL-2/ml) was added to the designated wells. Cultures were maintained at 37°C in a humidified 5% CO_2_ incubator for 72 hours. Cells were then harvested, stained, and analyzed by flow cytometry, or alternatively, [³H]-thymidine (18.5 kBq) was added for the final 8 or 16 hours of incubation before harvesting. For IL-2 titration studies, MACS-purified CD4^+^ CD25^+^ Tregs (5×10⁴ cells/well) and CTV-labeled CD8^+^ T cells (5×10⁴ cells/well) were co-cultured in anti-CD3 mAb-coated 96-well plates with titrated concentrations of IL-2 or IL-2/JES6.

### Toxicity studies

The toxicity of IL-2/JES6 and IL-2/S4B6 was evaluated by either 7 consecutive days injection of 2 µg IL-2/dose of each immunocomplex or 3 consecutive days injection of IL-2/S4B6 (2, 4 and 6 µg IL-2/dose) and IL-2/JES6 (8, 12 and 16 µg IL-2/dose). Body temperature (measured by stylus in the throat), body weight, and survival of mice were monitored.

### LEGENDplex Mouse Cytokine Release Syndrome Panel

Mice were i.p. injected on days 0, 1, and 2 with IL-2/JES6, Y33IC and control IC (2 µg eq IL-2/dose). On day 3, blood was collected via carotid bleeding. Serum was isolated and cytokine levels were measured using the LEGENDplex Mouse Cytokine Release Syndrome Panel (BioLegend, USA) according to the manufacturer’s protocol and analyzed by flow cytometry.

### Adoptive transfer mouse models

The spleen and lymph nodes were harvested from OT-I/Rag2^−/−^/Ly5.1 and OT-II/Rag1^−/−^/Ly5.1 and then single-cell suspensions were prepared in MACS buffer (PBS supplemented with 2 mM EDTA and 0.5% bovine serum albumin (BSA). CD8^+^ OT-I cells and CD4^+^ OT-II cells were purified by magnetic labeling of negative selection markers using a manual MACS separator (Miltenyi Biotec, Germany). 3×10^5^ purified CD8^+^ CD45.1^+^ OT-I and/or CD4^+^ CD45.1^+^ OT-II cells were adoptively transferred into acceptor C57BL/6 mice via tail vein injection in 200 µl PBS. Mice were then treated with 350 µg OVA or not via i.p. injection 1 day after transfer. ICIs (αCTLA-4 + αPD-1 antibodies; 0.5 mg/kg each) were injected i.p. on day 1 at least 6 hour before the IL-2co. They were then treated with IL-2co i.p. for 4 days. On day 5, the mice were sacrificed and their spleens were harvested and analyzed by flow cytometry.

In the experiments with the CD25 blockade, the adoptive transfer was performed as above except the mice were not injected with ICIs but i.p. with αCD25 mAb (200 µg/dose) on days 1 and 3 after adoptive transfer.

### Intracellular dye labeling

CTV dye was diluted in PBS at 0.5 µl/mL dilution. Then, purified CD8^+^ OT-I cells or CD4^+^ OT-II cells were resuspended in CTV solution as 4×10^6^ cells/mL. After 30 min of incubation at 37 ^ο^C, a huge excess of ice-cold PBS with 5% FBS was added. The stained cells were washed two times and diluted into 1.5×10^6^ cells/mL in PBS for adoptive transfer.

### Preparation of single-cell suspension for flow cytometry

For spleen cells, mice were sacrificed, and spleens were harvested and placed into gentleMACS C Tube. After spleens were homogenized by Gentle MACS Dissociator (Miltenyi Biotec, Germany) and red blood cells were lysed (ACK lysing buffer, Gibco, Gaithersburg, Maryland, USA). Cells were strained two times (70 µm and 30 µm) to remove clumps. Then resuspend in FACS buffer (PBS, 2% fetal calf serum (FCS), 2 mM EDTA).

For tumor cells, mice were sacrificed, and tumors were harvested without fat, fibrous, and necrotic areas. Tumors were placed into gentleMACS C tubes and cut into small pieces of 2–4 mm. Then cells were dissociated by mouse Tumor Dissociation Kit (Miltenyi Biotec) according to manufacturer instructions and resuspended in FACS buffer.

For blood cells, whole blood was collected into heparinized Eppendorf tubes via carotid bleeding of mice. Then blood was diluted 1:1 with FACS buffer and gently layered onto the same volume of histopaque-1083. Then cells were centrifuged at 800 g for 30 min at room temperature (RT) without brake. The upper plasma layer was discarded and peripheral blood mononuclear cells that are in the intermediate layer were harvested and washed two times with FACS buffer.

### Staining for surface and intracellular markers

Single cells suspended in spleen, tumor, or blood cells were blocked by 10% mouse serum and/or Fc block for 20 min on ice and stained with fluorochrome-labeled antibodies recognizing selected surface markers for 30 min on ice in the dark. After each step, cells were washed twice with FACS buffer and fixed with Foxp3 Fixation/Permeabilization buffer (eBiosciences, San Diego, California, USA) for 30 min on ice in the dark. After staining of surface antigens, fluorochrome-labeled antibodies recognizing intracellular markers were added and cells were incubated for 30 min on ice in the dark. Washing was performed twice with Fixation/Permeabilization buffer (eBiosciences, San Diego, California, USA). Cells were resuspended in FACS buffer before analysis. Flow cytometric analysis was performed on LSRII (BD Biosciences, San Jose, California, USA) and data were analyzed using FlowJo X software (Tree Star, Ashland, Oregon, USA). The gating strategy to identify immune cell subsets used for subsequent analyses is shown for splenocytes harvested from C57BL/6 mice in online supplemental figure 15.

### Tetramer staining

BALB/c mice were s.c. inoculated with 2×10^5^ CT26 cells on day 0. ICIs (αCTLA-4 + αPD-1 antibodies; 0.5 mg/kg each per dose) were i.p. injected on days 7, 10 and 13. Then mice were i.p. treated with IL-2/JES6 (8 µg IL-2/dose) on days 14, 15 and 16. On day 19, mice were sacrificed, and peripheral blood was collected into heparinized tubes via carotid artery bleeding. 10 µl of SPSYVYHQF/H-2Ld-APC tetramer and additional antibodies were prepared in 50 µl mix for each sample except fluorescence minus one control. 300 µl of blood was transferred onto 50 µl antibody mix and vortexed gently. After 30 min of incubation in the dark at room temperature, red blood cells were lysed by 1 mL ACK lysis buffer supplemented with 0.2% formaldehyde. Then, samples were vortexed immediately and incubated for 10 min in the dark at room temperature. Cells were washed two times with 3 mL FACS and centrifuged at 1300 rpm for 5 min. Lastly, cells were resuspended into 500 µl of PBS with 0.5% formaldehyde and analyzed by flow cytometry after 1 hour.

### Lung edema evaluation

C57BL/6 mice were treated with 2 µg equivalent IL-2/JES6, Y33 and control IC for 3 consecutive days. Mice were euthanized 1 day after the last dose and their lungs were harvested. Pulmonary wet weight was determined by weighing lungs before and after lyophilization overnight at 58°C under vacuum.

### Statistical analysis

Each experiment shown in main Figures was conducted a minimum of two times, yielding consistent results with n=6–20 technical replicates. Statistical analysis was carried out using GraphPad Prism (GraphPad Software, San Diego, California, USA). Group comparisons were assessed through unpaired two-tailed Student’s t-test, tumor growth analysis was conducted through one-way analysis of variance, with subsequent Dunnett’s post hoc test for comparison and a CI of 95% was applied. Survival analysis was performed using the Mantel-Cox log-rank test. Statistical significance was denoted as follows: *p≤0.05; **p≤0.01; ***p≤0.001.

## Supplementary material

10.1136/jitc-2024-010465 online supplemental file 1

10.1136/jitc-2024-010465 online supplemental file 2

10.1136/jitc-2024-010465 online supplemental file 3

10.1136/jitc-2024-010465 online supplemental file 4

10.1136/jitc-2024-010465 online supplemental file 5

10.1136/jitc-2024-010465 online supplemental file 6

10.1136/jitc-2024-010465 online supplemental file 7

10.1136/jitc-2024-010465 online supplemental file 8

10.1136/jitc-2024-010465 online supplemental file 9

10.1136/jitc-2024-010465 online supplemental file 10

10.1136/jitc-2024-010465 online supplemental file 11

10.1136/jitc-2024-010465 online supplemental file 12

10.1136/jitc-2024-010465 online supplemental file 13

10.1136/jitc-2024-010465 online supplemental file 14

10.1136/jitc-2024-010465 online supplemental file 15

10.1136/jitc-2024-010465 online supplemental file 16

10.1136/jitc-2024-010465 online supplemental file 17

## Data Availability

Data are available upon reasonable request.
