# Supplementary material for: Temporal optimization of CD25-biased IL-2 agonists and immune checkpoint blockade leads to synergistic anticancer activity despite robust regulatory T cell expansion
Source: J Immunother Cancer. 2025 Aug 11;13(8):e010465. doi: 10.1136/jitc-2024-010465 (PMC12352230; doi:10.1136/jitc-2024-010465)
Supplement: online supplemental file 1 [file jitc-13-8-s001.pdf]

Temporal optimization of CD25-biased IL-2 agonists and immune checkpoint blockade leads to synergistic anti-cancer activity despite robust regulatory T cell expansion

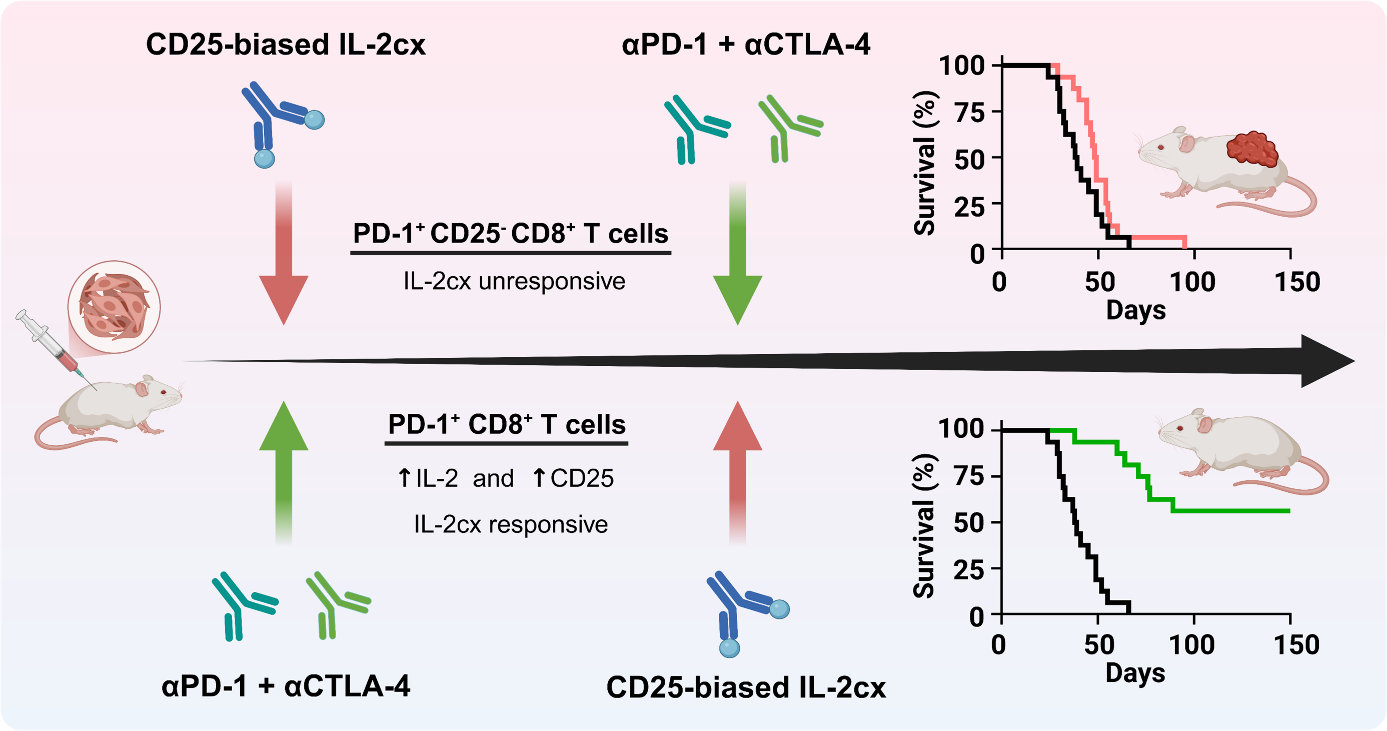

Authors

Irfan Baki Kilic, Petra Weberova, Derek VanDyke, Milada Sirova, Katerina Kubesova, Charina S. Fabilane, Vladyslav Mazhara, Kathy Liu, Katerina Behalova, Bohumil Ptacek, Blanka Rihova, Jamie B. Spangler and Marek Kovar

Correspondence

makovar@biomed.cas.cz

In Brief

- CD25-biased IL-2 complexes (IL-2cx) possess much lower toxicity than CD25-blocking ones, enabling a larger therapeutic window.
- CD25-biased IL-2cx selectively stimulate expansion and effector functions of activated $CD8^+$ T cells in a CD25-dependent manner and overcome Treg cell-mediated suppression.
- CD25-biased IL-2cx and immunocytokines synergize with ICIs to completely eradicate large, established tumors when IL-2cx are given simultaneously or after but not before ICIs
