# Supplementary figures and images for: Temporal optimization of CD25-biased IL-2 agonists and immune checkpoint blockade leads to synergistic anticancer activity despite robust regulatory T cell expansion
Source: J Immunother Cancer. 2025 Aug 11;13(8):e010465. doi: 10.1136/jitc-2024-010465 (PMC12352230; doi:10.1136/jitc-2024-010465)

# Online Supplemental Figure 1

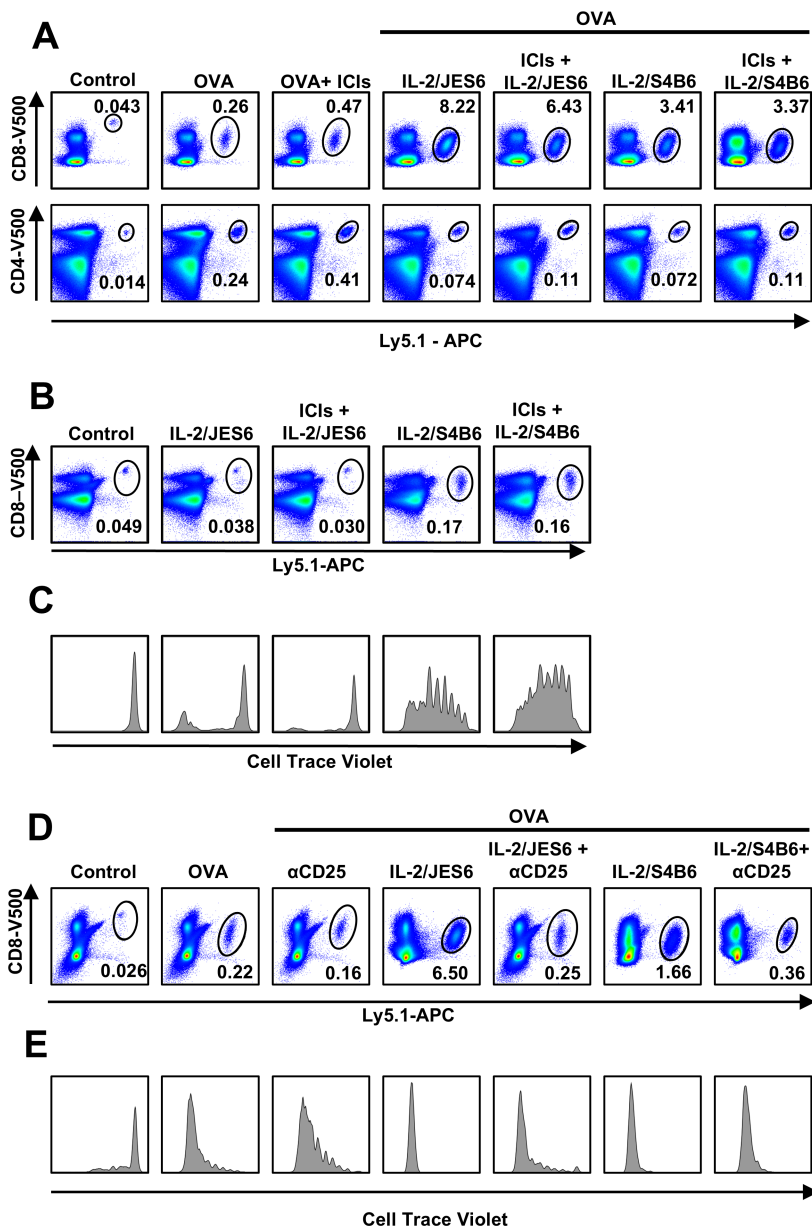

Supplement: online supplemental file 2 [file jitc-13-8-s002.pdf]

# Online Supplemental Figure 2

**A**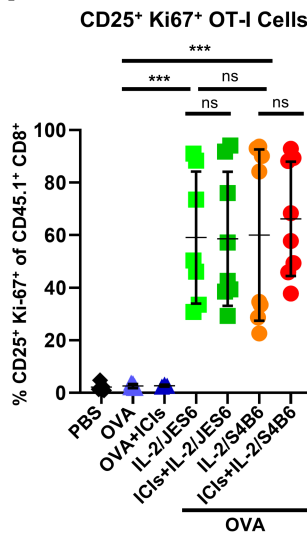**B**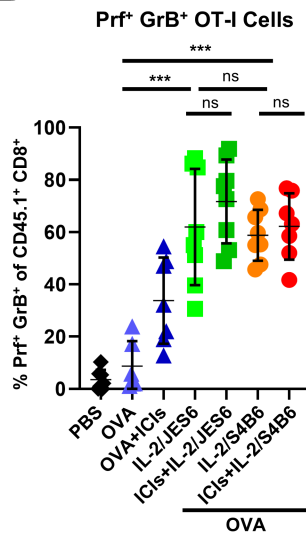**C**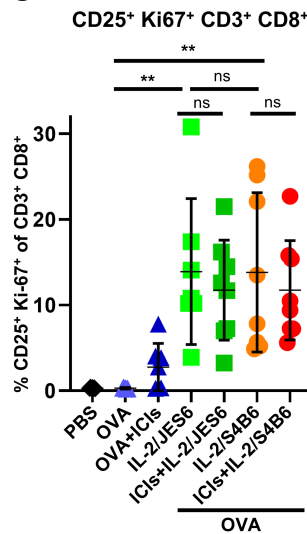**D**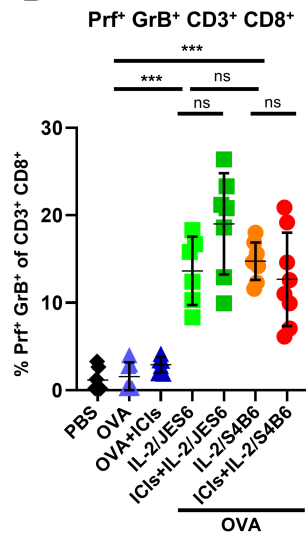**E**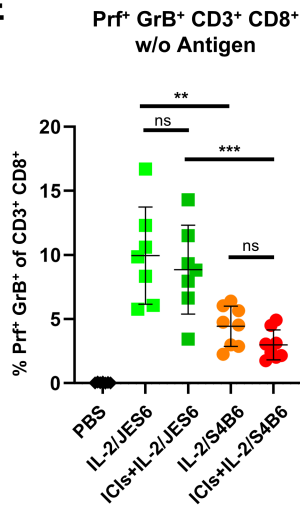**F**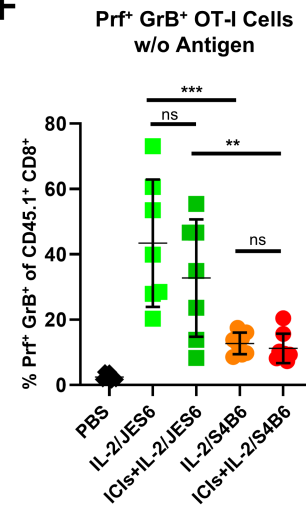**G**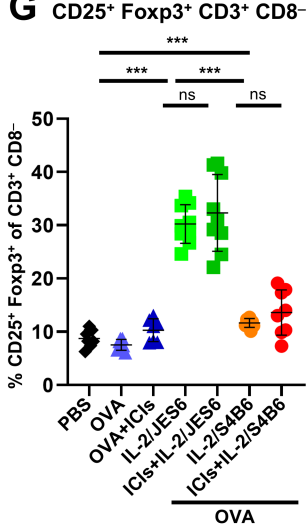**H**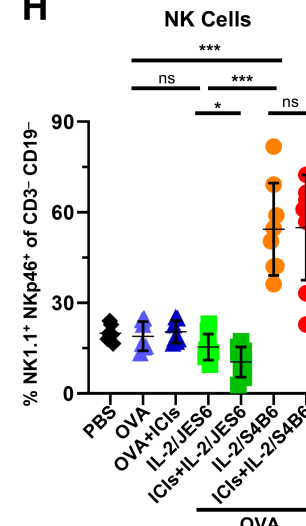

Supplement: online supplemental file 3 [file jitc-13-8-s003.pdf]

# Online Supplemental Figure 3

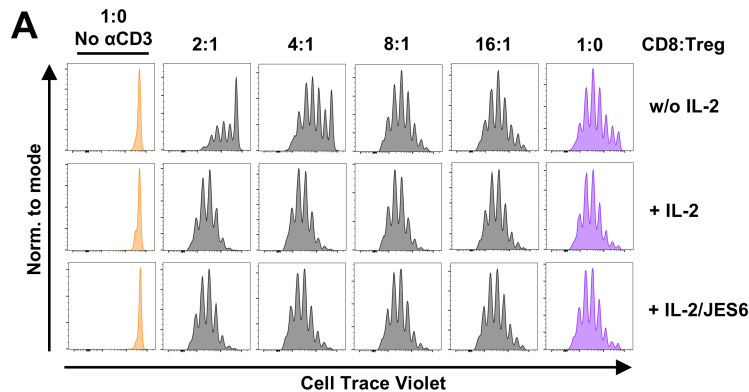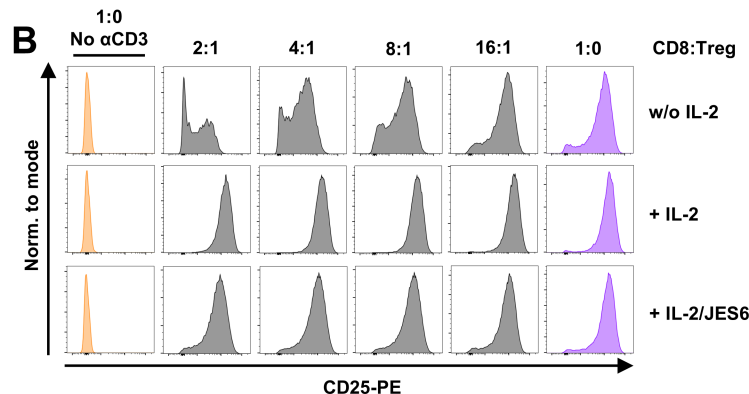

Supplement: online supplemental file 4 [file jitc-13-8-s004.pdf]

# Online Supplemental Figure 5

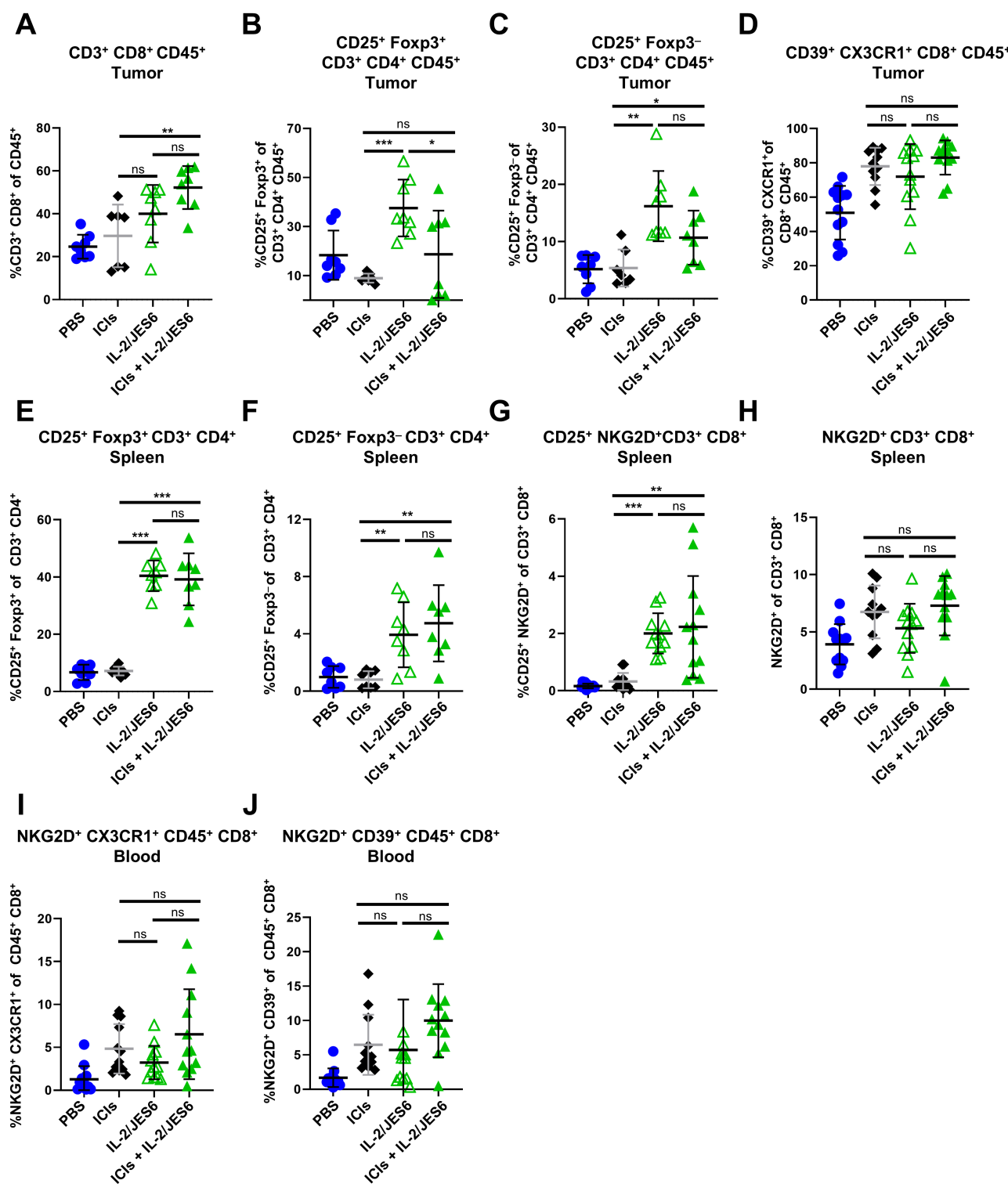

Supplement: online supplemental file 6 [file jitc-13-8-s006.pdf]

# Online Supplemental Figure 6

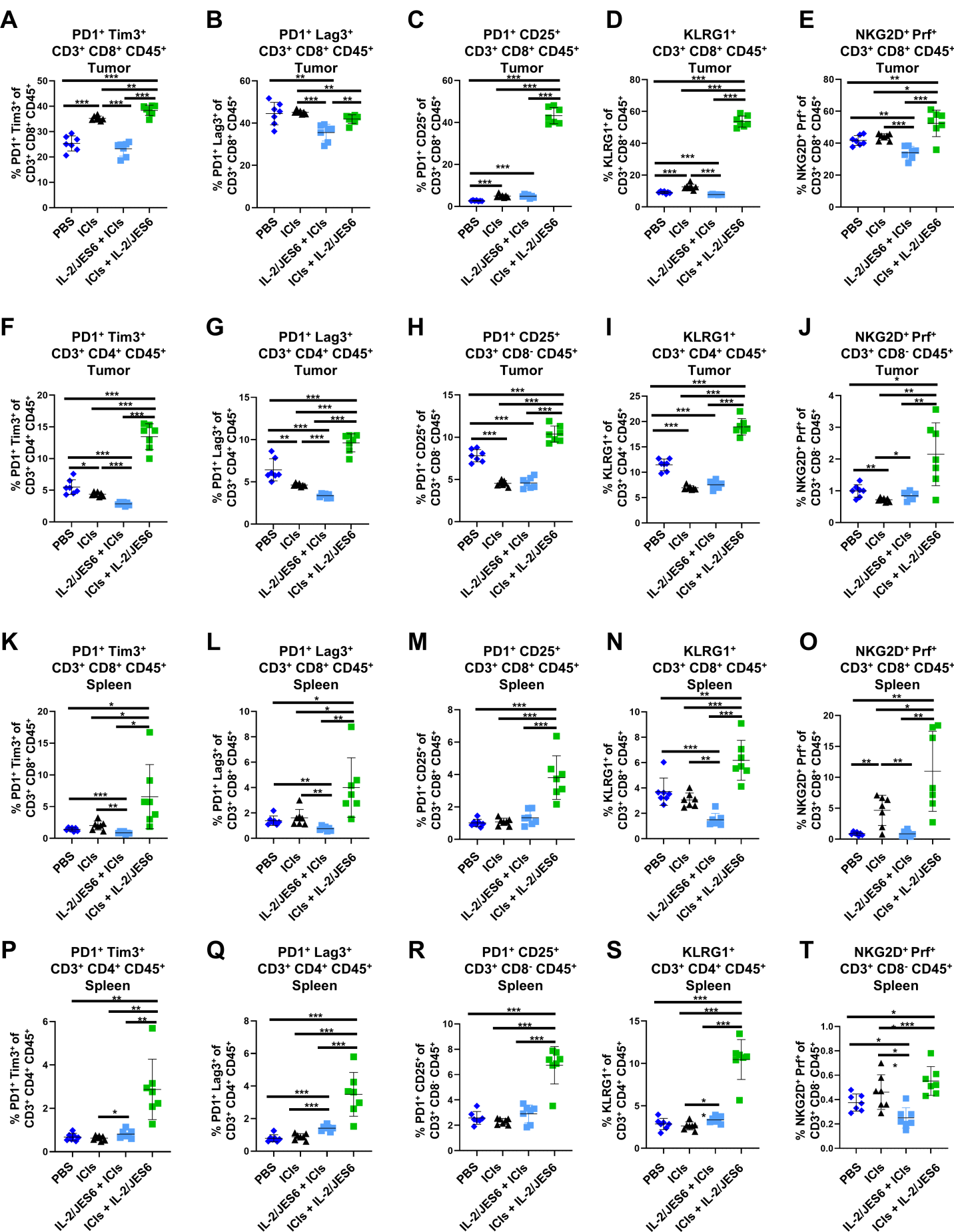

Supplement: online supplemental file 7 [file jitc-13-8-s007.pdf]

# Online Supplemental Figure 7

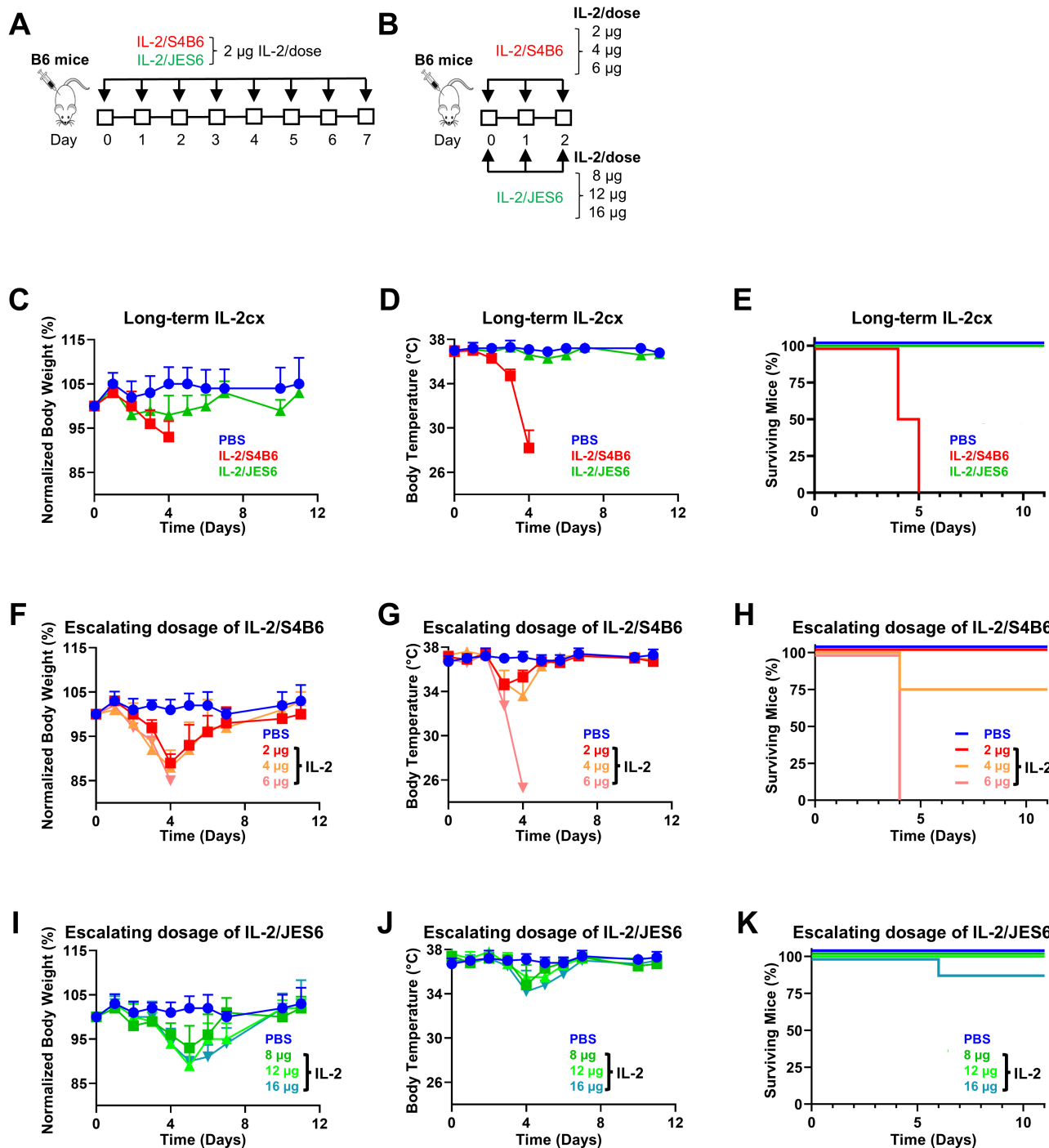

Supplement: online supplemental file 8 [file jitc-13-8-s008.pdf]

# Online Supplemental Figure 8

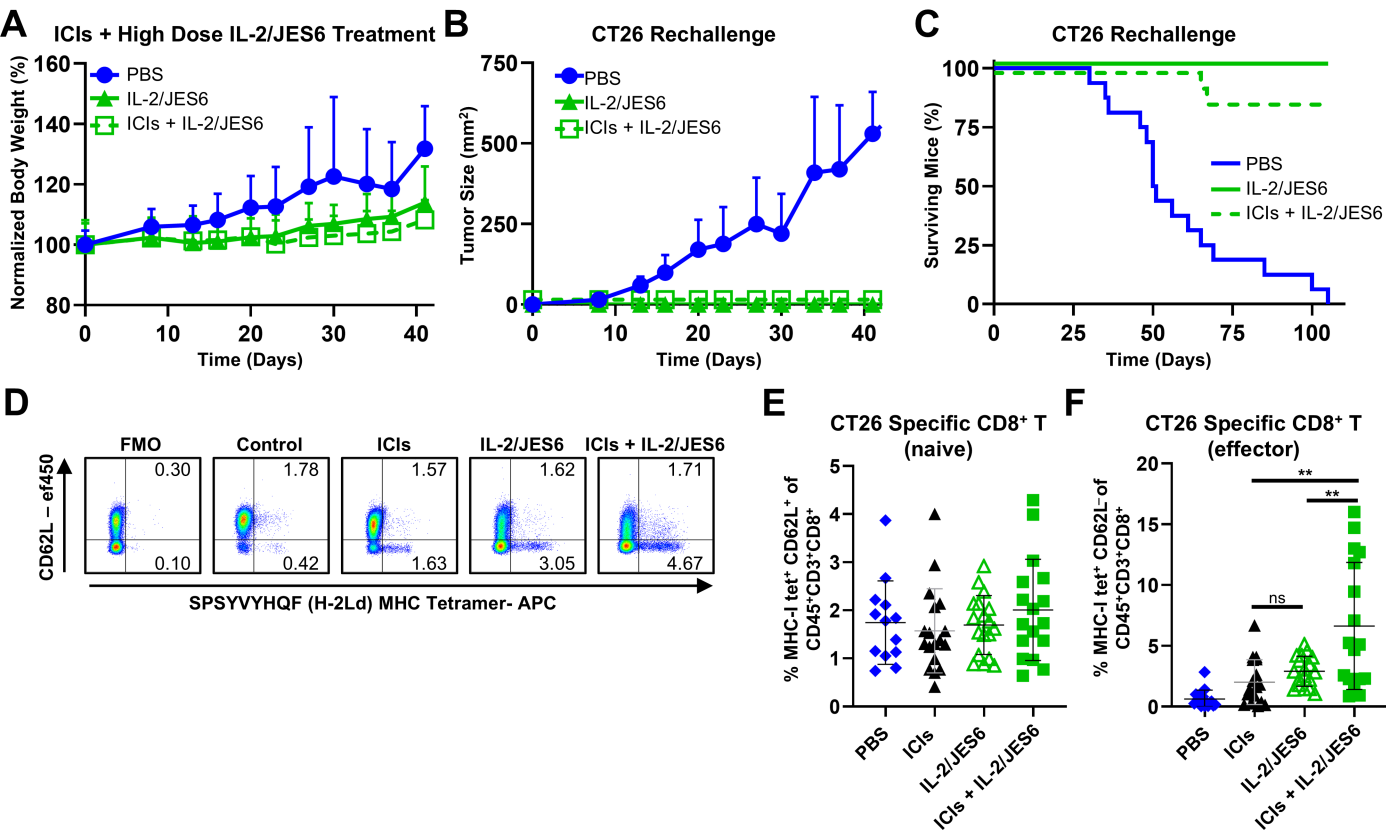

Supplement: online supplemental file 9 [file jitc-13-8-s009.pdf]

# Online Supplemental Figure 10

**A**

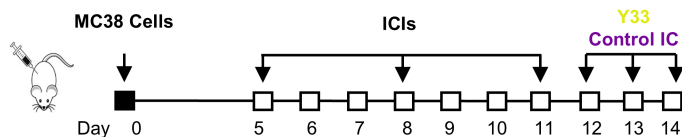

**B**

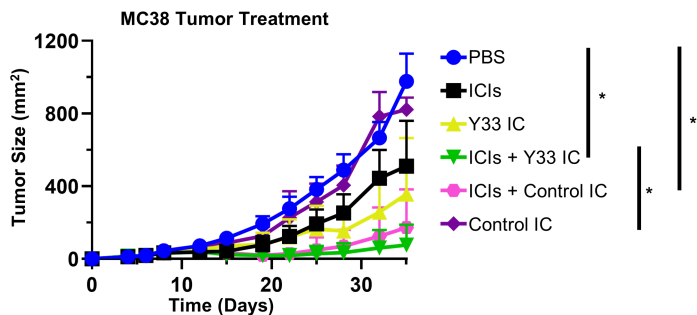

**C**

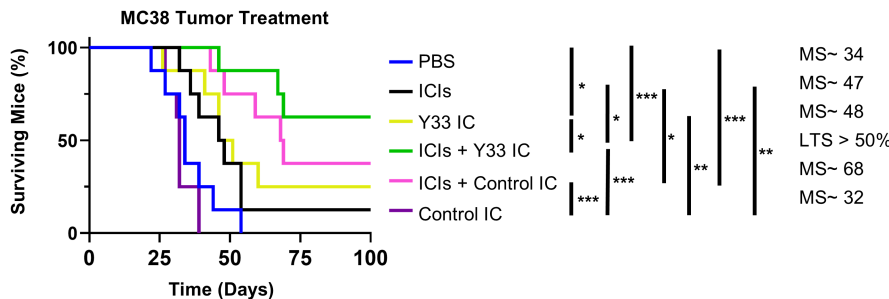

**D**

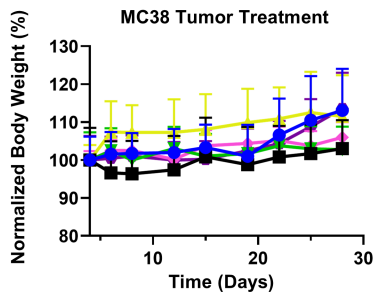

Supplement: online supplemental file 11 [file jitc-13-8-s011.pdf]

# Online Supplemental Figure 11

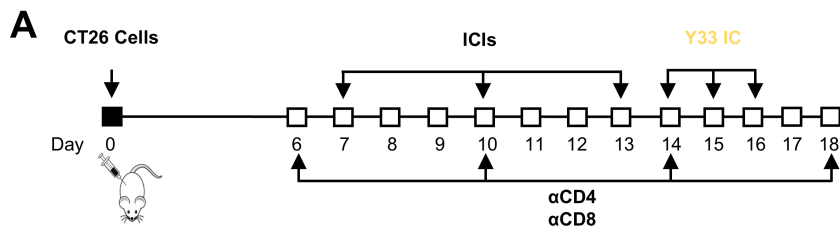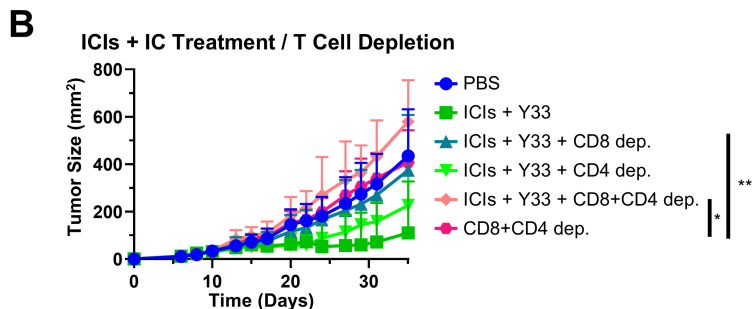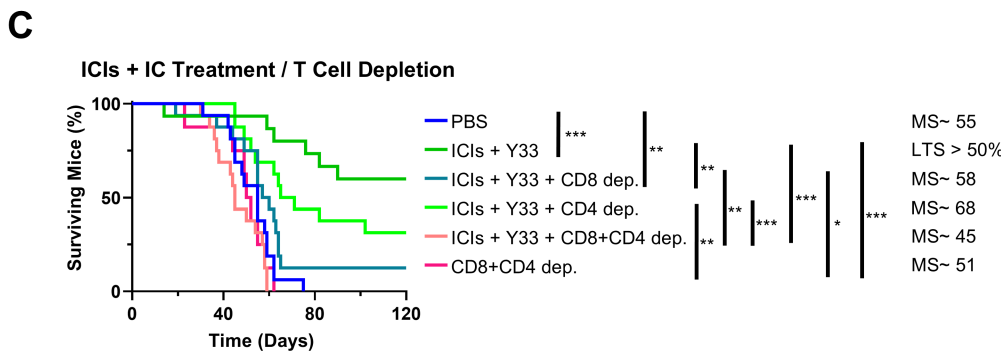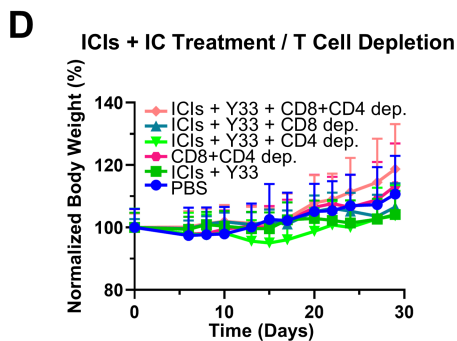

Supplement: online supplemental file 12 [file jitc-13-8-s012.pdf]

# Online Supplemental Figure 12

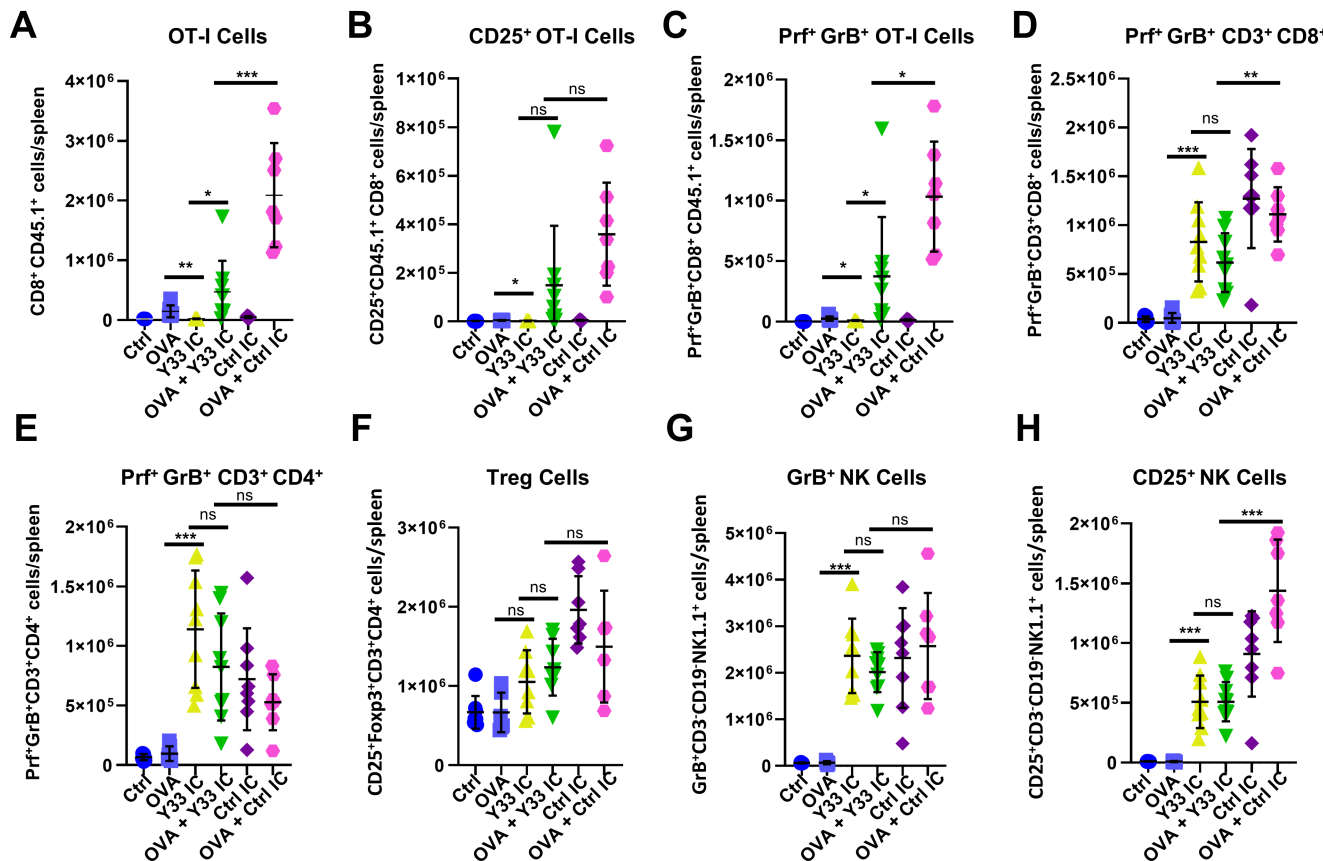

Supplement: online supplemental file 13 [file jitc-13-8-s013.pdf]

# Online Supplemental Figure 13

**A**

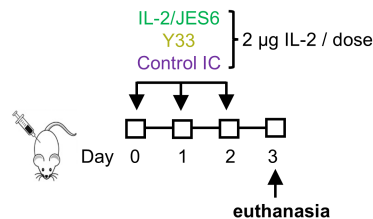

**B**

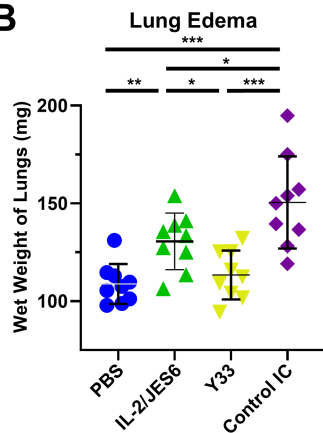

**C**

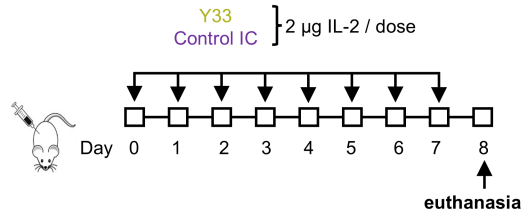

**D**

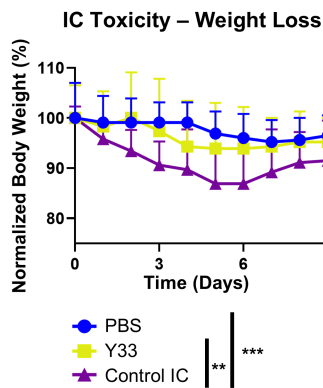

**E**

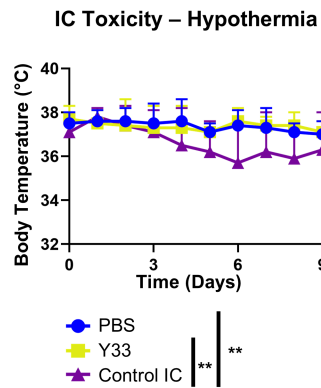

Supplement: online supplemental file 14 [file jitc-13-8-s014.pdf]

# Online Supplemental Figure 14

**A**

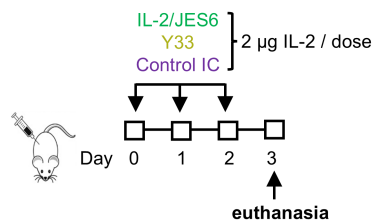

**B**

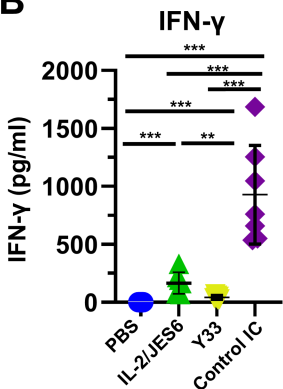

**C**

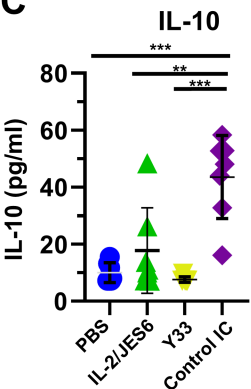

**D**

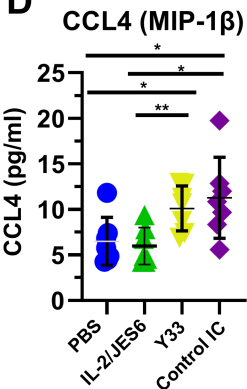

**E**

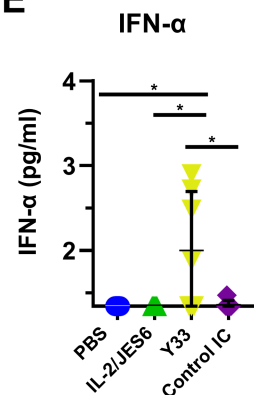

**F**

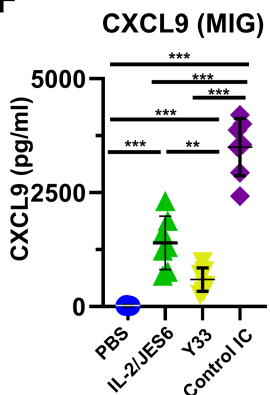

**G**

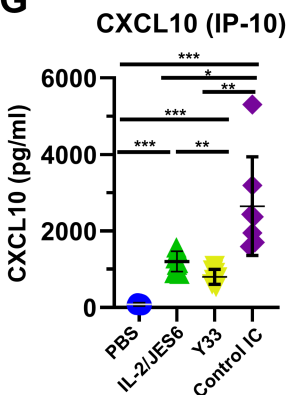

**H**

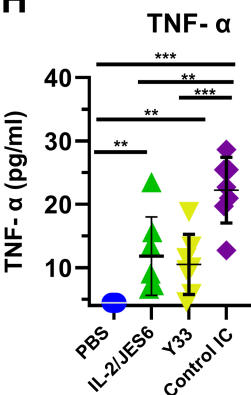

**I**

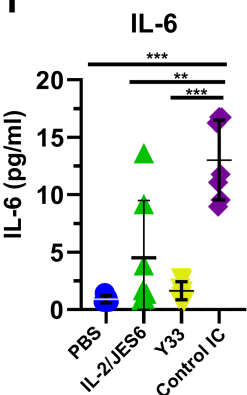

**J**

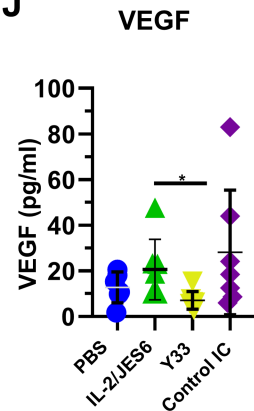

**K**

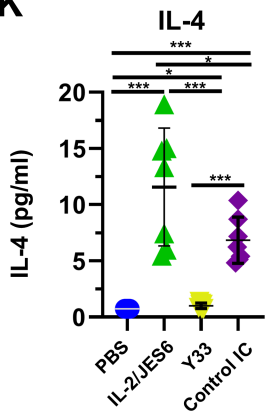

**L**

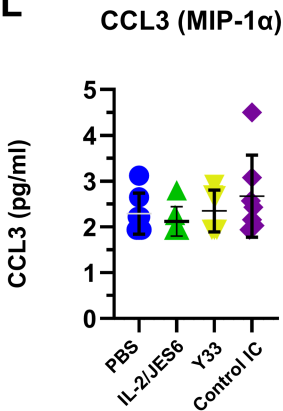

**M**

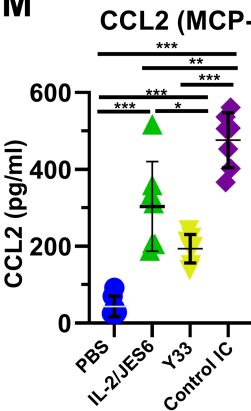

**N**

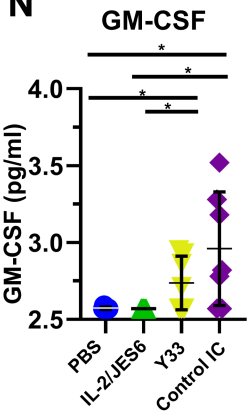

Supplement: online supplemental file 15 [file jitc-13-8-s015.pdf]

Online Supplemental Figure 15

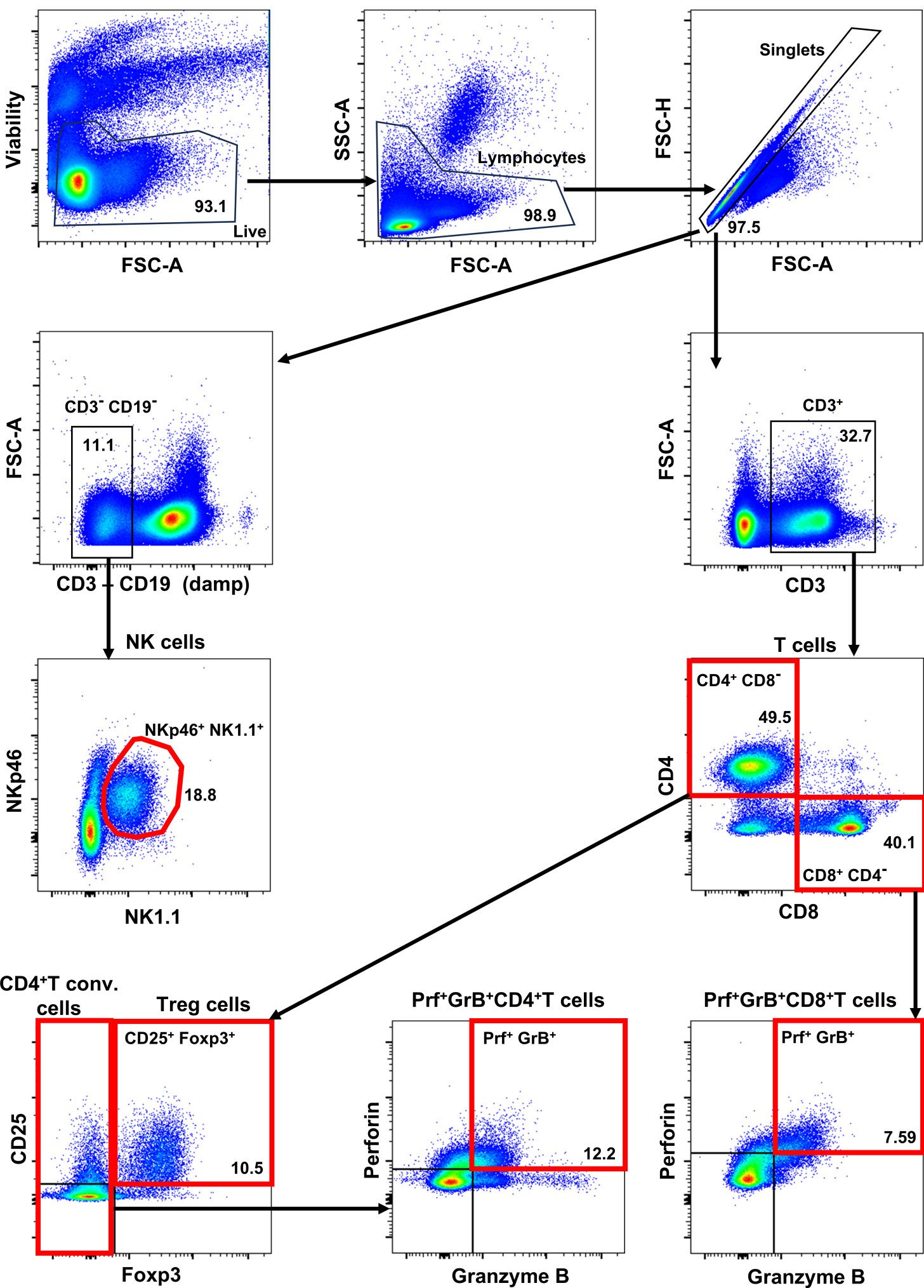

Supplement: online supplemental file 16 [file jitc-13-8-s016.pdf]
