# Supplementary material for: Temporal optimization of CD25-biased IL-2 agonists and immune checkpoint blockade leads to synergistic anticancer activity despite robust regulatory T cell expansion
Source: J Immunother Cancer. 2025 Aug 11;13(8):e010465. doi: 10.1136/jitc-2024-010465 (PMC12352230; doi:10.1136/jitc-2024-010465)
Supplement: online supplemental file 5 [file jitc-13-8-s005.pdf]

Online Supplemental Figure 4

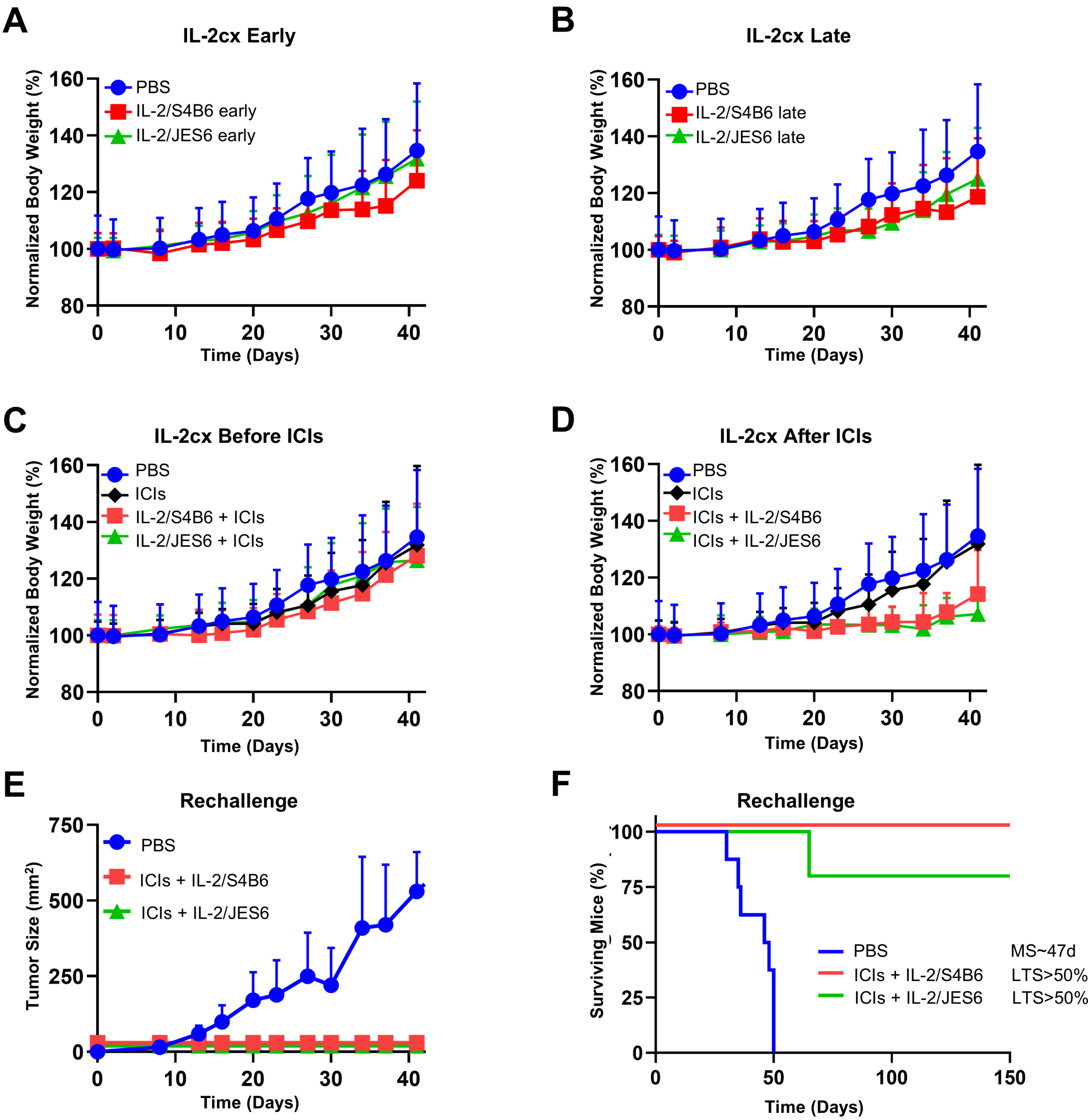

G

Percentage of long-term survivors

	PBS	ICIs	ICIs + IL-2/JES6	ICIs + IL-2/S4B6
Before	0%	12.5%	0%	0%
Simultenously	0%	20%	80%	50%
After	0%	12.5%	56.3%	31.3%
