# Supplementary material for: Temporal optimization of CD25-biased IL-2 agonists and immune checkpoint blockade leads to synergistic anticancer activity despite robust regulatory T cell expansion
Source: J Immunother Cancer. 2025 Aug 11;13(8):e010465. doi: 10.1136/jitc-2024-010465 (PMC12352230; doi:10.1136/jitc-2024-010465)

Online Supplemental Figure 9

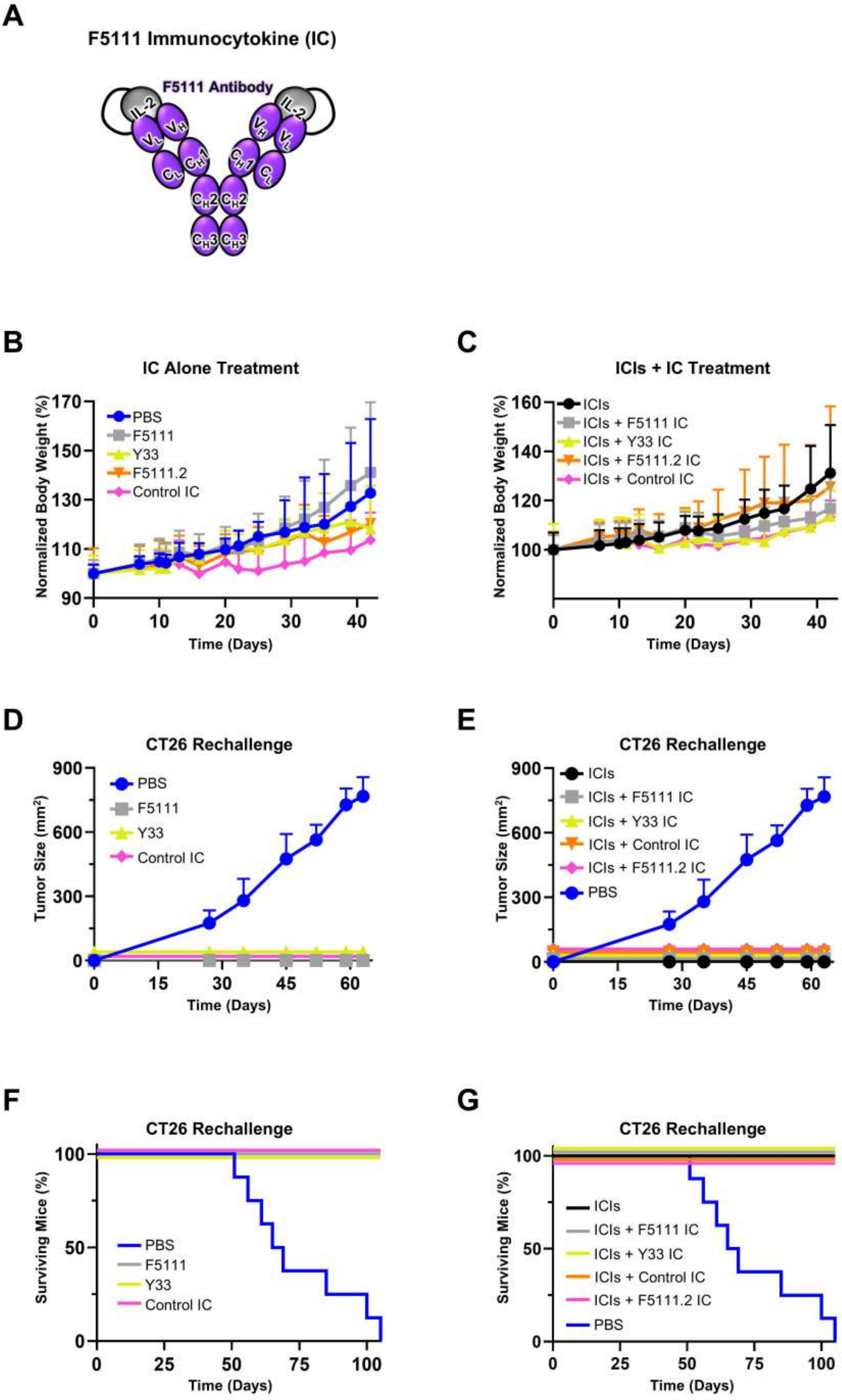

Supplement: online supplemental file 10 [file jitc-13-8-s010.pdf]
