# Supplementary material for: Temporal optimization of CD25-biased IL-2 agonists and immune checkpoint blockade leads to synergistic anticancer activity despite robust regulatory T cell expansion
Source: J Immunother Cancer. 2025 Aug 11;13(8):e010465. doi: 10.1136/jitc-2024-010465 (PMC12352230; doi:10.1136/jitc-2024-010465)
Supplement: online supplemental file 17 [file jitc-13-8-s017.pdf]

ONLINE SUPPLEMENTAL FIGURE LEGENDS

Online supplemental figure 1. IL-2/JES6 markedly expand antigen-primed CD8$^+$ T cells. A, Purified CD8$^+$ T cells or CD4$^+$ T cells from OT-I or OT-II/RAG1-/-/Ly5.1 mice, respectively, were adoptively transferred (AT) into B6 mice. Mice were i.p. injected with 350 μg OVA, ICIs (αCTLA-4 + αPD-1 antibodies; 0.5 mg/kg each) and IL-2co (2 μg IL-2/dose) and their spleens were analyzed by flow cytometry. Expansion of AT CD8$^+$ (**upper row**) and CD4$^+$ (**bottom row**) T cells in one representative mouse is shown for all experimental groups. **B-C**, Purified CTV-labelled OT-I CD8$^+$ T cells were AT into B6 mice. Treatment was the same as in (**A**) except the mice were not injected with OVA. Dot plots (**B**) and CTV profiles (**C**) showing expansion and proliferation of AT CD8$^+$ T cells in one representative mouse are presented for all experimental groups. **D, E**, Purified CTV-labelled OT-I CD8$^+$ T cells were AT into B6 mice. Treatment was the same as in (**A**) except the mice were not injected with ICIs but i.p. with αCD25 antibody (200 μg/dose) on days 1 and 3. Dot plots (**D**) and CTV profiles (**E**) showing expansion and proliferation of AT CD8$^+$ T cells in one representative mouse are presented for all experimental groups.

Online supplemental figure 2. IL-2/JES6 potently stimulate antigen-primed CD8$^+$ T cells and Treg cells but not NK cells. A-H, Purified CD8$^+$ T cells from OT-I or OT-II/RAG1$^{-/-}$/Ly5.1 mice were adoptively transferred (AT) into B6 mice. Mice were i.p. injected with 350 μg OVA (except **E**, **F**, where OVA was not injected), ICIs (αCTLA-4 + αPD-1 antibodies; 0.5 mg/kg each), and IL-2 complexes (2 μg IL-2/dose) and their spleens were analyzed by flow cytometry. The treatment scheme was as described in Figure 1 A. Expression of various markers in AT OT-I CD8$^+$ T cells (**A, B, F**) and endogenous CD8$^+$ T cells (**C, D, E**), expansion of Treg cells (**G**), and expansion of NK cells (**H**) are presented. Results are shown as average ± SD for all experimental groups and each point represents an individual mouse. Data were pooled from

2 independent experiments with n = 5-8. Results were statistically analyzed by unpaired t-test (*: $P < 0.05$; **: $P < 0.01$; ***: $P < 0.001$).

Online supplemental figure 3. IL2/JES6 overcome Treg cell mediated suppression of CD8$^+$ T cells. A-B, CTV-labelled CD8$^+$ T cells were co-cultivated with Treg cells at the indicated ratios in αCD3 antibody coated wells alone, with IL 2, or with IL 2/JES6 (10 ng IL 2/ml) for 72 h. Histograms of CTV profiles (**A**) and CD25 expression in CD8$^+$ T cells (**B**) are shown for each experimental condition. Experiments were performed 3 times with similar results.

Online supplemental figure 4. IL-2cx, either alone or in combination with ICIs, provide long-term immunity against CT26 tumors without inducing toxicity. A-G, BALB/c mice were s.c. inoculated with 2×10^5 CT26 cells on day 0. Mice were i.p. injected with ICIs (αCTLA-4 + αPD-1 antibodies; 0.5 mg/kg each per dose), IL-2cx (2 μg IL-2/dose), or both. Control mice were i.p. injected with the same volume (250 μL) of PBS. Schema of the treatment was as described in Figure 3A. IL-2cx were administered on days 4, 5 and 6 (early) (**A**) or on days 14, 15 and 16 (late) (**B**). Alternatively, IL-2cx were administered before treatment with ICIs (**C**) or after treatment with ICIs (**D**). Body weight of mice (**A-D**) was monitored. Each experimental point represents average ± SD. Data were pooled from 2 independent experiments with n = 16 for each experimental group. Long-term surviving (LTS) mice cured of CT26 tumors with ICIs + IL-2/JES6 or ICIs + IL-2/S4B6 were s.c. re-challenged with 2×10^5 CT26 cells on day 150 post primary CT26 tumor cell inoculation. Naïve BALB/c mice were used as a control. Tumor growth (**E**) and survival of mice (**F**) were monitored. Each experimental point represents average ± SD. Data were pooled from 2 independent experiments with control: n = 8; ICIs +

IL-2/S4B6: n = 5; ICIs + IL-2/JES6: n = 9. Mean survival (MS) is shown for each experimental group, except in cases where the proportion of LTS mice exceeded 50%. Comprehensive table showing the percentage of long-term surviving mice within various treatment schedules, where IL-2cx were administered either before, simultaneously with, or after ICIs (G).

Online supplemental figure 5. IL-2/JES6 in combination with ICIs leads to robust accumulation of CD8$^+$ T cells with activated/effector phenotype within the tumor. A-I, BALB/c mice were s.c. inoculated with 2×10^5 CT26 cells on day 0. Mice were i.p. injected with ICIs (αCTLA-4 + αPD-1 antibodies; 0.5 mg/kg each per dose; days 10, 13 and 16), IL-2/JES6 (2 μg IL-2/dose; days 17, 18 and 19), or both. Control mice were i.p. injected with the same volume (250 μL) of PBS. Mice were euthanized on day 21. Blood, spleens, and tumors were harvested, and various immune cell subsets were analyzed by flow cytometry (**A-J**). Results are shown as average ± SD for all experimental groups and each point represents an individual mouse. Data were pooled from 3 independent experiments with n = 10-12. Results were statistically analyzed by unpaired t-test (*: $P < 0.05$; **: $P < 0.01$; ***: $P < 0.001$).

Online supplemental figure 6. IL-2/JES6 given after but not before ICIs leads to enhanced expression of terminal differentiation markers as well as effector function markers on T cells in tumor and spleen. BALB/c mice were s.c. inoculated with 2×10^5 CT26 cells on day 0. Mice were then i.p. injected with ICIs (αCTLA-4 + αPD-1 antibodies; 0.5 mg/kg each per dose; days 10, 13 and 16) alone and with ICIs either preceded or followed by administration of IL-2/JES6 (2 μg IL-2/dose; days 7, 8 and 9 or 17, 18 and 19, respectively). Control mice were i.p. injected with an equivalent volume (250 μL) of PBS. Mice were euthanized on day 21. Tumors and spleens were harvested and analyzed by flow cytometry to determine phenotype of CD8$^+$

and $CD4^+$ T cells (A-T). Results are shown as average ± SD for all experimental groups and each point represents an individual mouse. Data shown are from one experiment with n = 7. Results were statistically analyzed by unpaired t-test (*: P < 0.05; **: P < 0.01; ***: P < 0.001).

Online supplemental figure 7. IL-2/JES6 are substantially less toxic than IL-2/S4B6. A-K, BALB/c mice were i.p. injected with IL-2/JES6 or IL-2/S4B6 using either daily administration of the same dose for 8 days (**A**) or titrated dosage of complexes given daily for 3 days (**B**). Control mice were i.p. injected with the same volume (250 µL) of PBS. For **c-e**, IL-2 complexes were administered as shown in (**A**), and for **F-K,** IL-2 complexes were administered as shown in (**B**). Body weight (**C**, **F**, **I**), body temperature (**D**, **G**, **J**) and survival (**E**, **H**, **K**) for each mouse were recorded. Data pooled from 2 independent experiments with n = 8 for each experimental group. Data are presented as average ± SD.

Online supplemental figure 8. High-dose IL-2/JES6, either alone or in combination with ICIs, provide powerful long-term immunity against CT26 tumors without inducing toxicity. A-C, BALB/c mice were s.c. inoculated with 2×10^5 CT26 cells on day 0. Mice were i.p. injected with ICIs (αCTLA-4 + αPD-1 antibodies; 0.5 mg/kg each per dose), IL-2 complexes (8 µg IL-2/dose), or both. Control mice were i.p. injected with the same volume (250 µL) of PBS. Schema of the treatment was as described in Figure 5A. Body weight of mice was monitored (**A**). Each experimental point represents average ± SD. Data were pooled from 2 independent experiments with n = 16 for each experimental group. Long-term surviving (LTS) mice cured of CT26 tumors with IL-2/JES6 or ICIs + IL-2/JES6 were s.c. re-challenged with 2×10^5 CT26 cells on day 150 post primary CT26 tumor cell inoculation. Naïve BALB/c mice were used as a control. Tumor growth (**B**) and survival of mice (**C**) were monitored. Each

experimental point represents average ± SD. Data were pooled from 2 independent experiments with control: n = 16; ICIs + IL-2/JES6: n = 15; IL-2/JES6: n = 6. Mean survival (MS) is shown for each experimental group, except in cases where the proportion of LTS mice exceeded 50%. **D-F,** BALB/c mice were inoculated with CT26 cells and treated with ICIs and IL-2/JES6, as in Figure 5A. Mice were euthanized on day 19. Peripheral blood cells were stained for CD62L and with SPSYVYHQF/H-2L^d-APC or PE tetramer and analyzed by flow cytometry. Tetramer$^+$CD62L$^+$ and tetramer$^+$CD62L$^-$ cells (%) in CD8$^+$ T cells from one representative mouse (**D**) and with depicted average ± SD for all experimental groups (**E**, **F**) are shown. Each experimental point represents average ± SD. Data were pooled from 3 independent experiments with n = 15-20. Results were statistically analyzed by unpaired t-test (**: P < 0.01).

Online supplemental figure 9. CD25-biased single-chain ICs, either alone or in combination with ICIs, provide long-term immunity against CT26 tumors without inducing toxicity. A, Schematic of the F5111 immunocytokine (IC), which fuses the human IL-2 cytokine to the full-length F5111 antibody. Heavy chain and light chain variable (variable heavy (V_H) and variable light (V_L)) and constant domains (heavy chain constant domains 1, 2, and 3 (C_H1, C_H2, and C_H3, respectively) and light chain constant domain (C_L)) are indicated. **B-E,** BALB/c mice were s.c. inoculated with 2×10^5 CT26 cells on day 0. Mice were i.p. injected with ICIs (αCTLA-4 + αPD-1 antibodies; 0.5 mg/kg each per dose), hIL-2-based ICs (2 μg IL-2/dose), or combined ICIs+IC therapies. Control mice were i.p. injected with the same volume (250 μL) of PBS. Schema of the treatment was as described in Figure 6A. Body weight of mice was monitored (**B, C**). Each experimental point represents average ± SD. Body weight is shown for 1 out of 2 independent experiments with n = 8 for all experimental groups. Long-term surviving (LTS) mice cured of CT26 tumors with IC alone or ICIs + IC were s.c. re-challenged with 2×10^5 CT26 cells on day 150 post primary CT26 tumor cell inoculation. Naïve BALB/c

mice were used as a control. Tumor growth (**D**, **E**) and survival of mice (**F**, **G**) were monitored. Each experimental point represents average ± SD. Data were pooled from 2 independent experiments with control: n = 8; F5111: n = 5; Y33: n = 5; control IC: n = 3; ICIs: n = 3; ICIs + F5111: n = 11; ICIs + Y33: n = 15; ICIs + F5111.2: n = 7; ICIs + control IC: n = 10.

Online supplemental figure 10. Y33 IC potentiates the antitumor activity of ICIs in MC38 mouse model of cancer. A-D, B6 mice were s.c. inoculated with 1×10^6 MC38 cells on day 0. Mice were i.p. injected with ICIs (αCTLA-4 + αPD-1 antibodies; 0.5 mg/kg each per dose), human IL-2-based ICs (2 µg IL-2/dose), or combined ICIs+IC therapies. Control mice were i.p. injected with the same volume (250 µL) of PBS. A schematic of the study is shown (**A**). Weight (**B**), tumor growth (**C**), and survival of mice (**D**) were monitored. Each experimental point represents average ± SD. The experiment was performed once with n = 8 for each experimental group. Tumor growth was analyzed by one-way ANOVA, followed by Dunnett's post-hoc test. Survival was analyzed by the Mantle-Cox log-rank test (*: $P < 0.05$; **: $P < 0.01$; ***: $P < 0.001$). Mean survival (MS) is shown for each experimental group, except in cases where the proportion of long-term surviving (LTS) mice exceeded 50%.

Online supplemental figure 11. Antitumor activity of combined treatment with Y33 IC and ICIs is predominantly mediated by $CD8^+$ T cells. A-D, BALB/c mice were s.c. inoculated with 2×10^5 CT26 cells on day 0. Mice were i.p. injected with ICIs (αCTLA-4 + αPD-1 antibodies; 0.5 mg/kg each per dose) and Y33 IC (2 µg IL-2/dose). Control mice were i.p. injected with the same volume (250 µL) of PBS. Some experimental groups were injected with αCD4 antibody (150 µg/dose), αCD8 antibody (150 µg/dose), or both antibodies. A schematic of the study is shown (**A**). Tumor growth (**B**), survival of mice (**C**), and body weight (**D**) were monitored. Each experimental point represents average ± SD. Pooled data from 2 independent

experiments (n = 16) are shown. Tumor growth was analyzed by one-way ANOVA, followed by Dunnett's post-hoc test. Survival was analyzed by the Mantle-Cox log-rank test (*: P < 0.05; **: P < 0.01; ***: P < 0.001). Mean survival (MS) is shown for each experimental group, except in cases where the proportion of long-term surviving (LTS) mice exceeded 50%.

Online supplemental figure 12. Y33 IC expands antigen-primed CD8$^+$ T cells and significantly boosts their expression of effector molecules. A-H, Purified CD8$^+$ T cells from OT-I/RAG1$^{-/-}$/Ly5.1 mice were adoptively transferred (AT) into C57BL/6 mice. Mice were i.p. injected with 350 μg OVA, hIL-2-based ICs (2 μg IL-2/dose), or OVA+ICs, and their spleens were analyzed by flow cytometry as shown in fig. 7A. Absolute counts of various lymphocyte populations in the spleen are shown as an average ± SD for each experimental group. Each point represents an individual mouse. Data were pooled from 2 independent experiments with n = 6-9 for each experimental group. Results were statistically analyzed by unpaired t-test (*: P < 0.05; **: P < 0.01; ***: P < 0.001).

Online supplemental figure 13. Y33IC is significantly less toxic in comparison to control IC. A-B, BALB/c mice were i.p. injected with IL-2/JES6, Y33 IC or control IC (2 μg IL-2/dose) daily for 3 days. Control mice were i.p. injected with the same volume (250 μL) of PBS. A schematic of the study is shown (**A**). Mice were euthanized and the wet weight of lungs was determined (**B**). Each experimental point represents average ± SD. Data were pooled from 2 independent experiments with n = 9 for each experimental group. Results were statistically analyzed by unpaired t-test (*: P < 0.05; **: P < 0.01; ***: P < 0.001). **C-E,** BALB/c mice were i.p. injected with Y33 IC or control IC (2 μg IL-2/dose) daily for 8 days. Control mice were i.p. injected with the same volume (250 μL) of PBS. A schematic of the study is shown (**C**). Body weight (**D**) and body temperature (**E**) were recorded. Each experimental point represents

average ± SD. Data were pooled from 2 independent experiments with n = 8 for each experimental group. Results were statistically analyzed by one-way ANOVA, followed by Tukey's post-hoc test (**: $P < 0.01$; ***: $P < 0.001$).

Online supplemental figure 14. IL-2/JES6 and Y33 IC do not induce cytokine storm, whereas native IL-2-based Control IC elicits significant cytokine secretion. A, B6 mice were i.p. injected with IL-2/JES6, Y33 IC, or Control IC (2 μg IL-2/dose) daily for 3 days. Mice in the control group were injected with an equivalent volume (250 μL) of PBS. A schematic of the study is shown (**A**). **(B-N)** Blood samples were collected from mice on day 3 after the treatment and cytokine levels in the sera were determined using the LEGENDplex™ Mouse Cytokine Release Syndrome Panel. IFN-γ **(B)**, IL-10 **(C)**, CCL4 **(D)**, IFN-α **(E)**, CXCL9 **(F)**, CXCL10 **(G)**, TNF- α **(H)**, IL-6 **(I)**, VEGF **(J)**, IL-4 **(K)**, CCL3 **(L)**, CCL2 **(M)**, and GM-CSF **(N)** levels are shown. n = 7 for each group. The results were analyzed using unpaired t-test (*: $P < 0.05$; **: $P < 0.01$; ***: $P < 0.001$).

Online supplemental Figure 15. Flow cytometry immune cell subset gating strategy for splenocytes from C57BL/6 mice. All subsets were derived from the Live gate> Lymphocytes> Singlets. Within this gate, NK cells were gated as $CD3^{-}CD19^{-}$> $NK1.1^{+}NKp46^{+}$ cells, $CD4^{+}$ Tconv cells were gated as $CD3^{+}$> $CD4^{+}CD8^{-}$> $FoxP3^{-}$ cells, $CD8^{+}$ T cells were gated as $CD3^{+}$> $CD4^{-}CD8^{+}$ cells, and Treg cells were gated as $CD3^{+}$> $CD4^{+}CD8^{-}$> $CD25^{+}FoxP3^{+}$ cells.
